# Measuring income for catastrophic cost estimates: Limitations and policy implications of current approaches

**DOI:** 10.1016/j.socscimed.2018.08.041

**Published:** 2018-10

**Authors:** Sedona Sweeney, Rachel Mukora, Sophie Candfield, Lorna Guinness, Alison D. Grant, Anna Vassall

**Affiliations:** a TB Centre, London School of Hygiene & Tropical Medicine, London, UK; b The Aurum Institute, Johannesburg, South Africa; c Africa Health Research Institute, School of Nursing and Public Health, University of KwaZulu-Natal, South Africa; d School of Public Health, University of the Witwatersrand, Johannesburg, South Africa

**Keywords:** South Africa, Catastrophic cost, Coping, Tuberculosis, Methods, Income

## Abstract

There is increasing global policy interest in estimating catastrophic costs incurred by households because of ill health, and growing need for information on disease-specific household cost data. There are several methodological approaches used to estimate income and no current consensus on the best method for estimating income in the context of a survey at the health facility. We compared six different approaches to estimate catastrophic cost among patients attending a health facility in South Africa. We used patient cost and income data collected June 2014–March 2015 from 66 participants enrolled in a clinical trial in South Africa (TB FastTrack) to explore the variation arising from different income estimation approaches and compared the number of households encountering catastrophic costs derived for each approach. The total proportion of households encountering catastrophic costs varied from 0% to 36%, depending on the estimation method. Self-reported mean annual income was significantly lower than permanent income estimated using an asset linking approach, or income estimated using the national average. A disproportionate number of participants adopting certain coping strategies, including selling assets and taking loans, were unable to provide self-reported income data. We conclude that the rapid methods for estimating income among patients attending a health facility are currently inconsistent. Further research on methods for measuring income, comparing the current recommended methods to ‘gold standard’ methods in different settings, should be done to identify the most appropriate measurement method.

## Highlights

• Rapid methods for estimating income for catastrophic TB costs are inconsistent.
• Depending on income estimation approach, 0–36% households faced catastrophic TB costs.
• Quantitative indicators of catastrophe did not reflect extent of coping strategy use.

## 1 Introduction

Costs incurred as a result of ill-health can aggravate household vulnerability (Alam and Mahal, 2014; Wagstaff and Lindelow, 2014). They can also contribute to delays in diagnosis, reduced adherence, and poorer health outcomes (Wingfield et al., 2014). Tuberculosis (TB) patients often encounter substantial costs in the form of out-of-pocket payments and lost income. In recognition of the impact of these costs, the End TB Strategy introduced a TB-specific indicator of financial risk protection; this is labelled “catastrophic total costs due to TB”, and includes medical and non-medical direct costs and income losses (Lönnroth et al., 2014). The End TB Strategy targets specify that no patient encounters catastrophic total costs due to TB by the year 2020 (World Health Organization, 2015).

The indicator of ‘total catastrophic costs due to TB’ is relatively new and requires a different measurement approach and definition of ‘catastrophic’ compared to that used for general catastrophic health expenditure measured in the context of health financing. This paper aims to inform guidance on the measurement of catastrophic total costs due to TB from a sample of patients interviewed as part of a facility-based survey. We compare estimates of the prevalence of catastrophic cost using six approaches. We highlight the implications of these measurement approaches on the identification of catastrophic costs and resulting policy.

### 1.1 Background

To support countries seeking to meet the target of zero catastrophic costs due to TB by 2020 (World Health Organization, 2015), the World Health Organisation (WHO) TB Programme established a Task Force in 2015 to develop a generic protocol for estimating the prevalence of catastrophic costs, building on methods used in previous studies of patient costs to provide guidance to countries on estimating catastrophic cost (World Health Organization, 2017a). The aim of the ‘catastrophic total cost’ measure as described in the WHO handbook is to capture where health-related costs are likely to have a substantial impact on the household's ability to pay for basic subsistence needs; this is represented in terms of total costs as a proportion of household capacity to pay. For global monitoring of the End TB Strategy catastrophic cost indicator, the WHO has chosen to use a threshold of 20% of annual household income. This threshold is currently used by National TB Programmes (NTP) implementing the WHO survey for annual reports to WHO (World Health Organization, 2017b), however countries are also encouraged to undertake sensitivity analyses around the threshold.

In the context of health financing, the numerator for the “catastrophic expenditures” equation has been traditionally measured as direct out-of-pocket expenditure (Xu et al., 2005). However, over half of the economic burden encountered by households during an episode of TB comes in the form of lost income and lost productivity due to illness or time spent care-seeking (indirect costs) (Tanimura et al., 2014). The indicator of ‘catastrophic costs due to TB’ therefore includes indirect costs. Indirect costs are most commonly estimated through two approaches: first, household income can be estimated before and after the TB episode; any direct income loss due to TB is then captured by taking the difference. Second, the number of hours spent seeking care or otherwise unable to work due to TB can be estimated, and the value of these hours approximated with an estimate of the earning capacity of the patient for that time (e.g. hourly income). The first approach captures only the loss of paid work, while the second approach captures all time off work necessitated by symptoms and treatment seeking (but may not include any household mitigation of that loss).

There are several potential indicators of household capacity to pay for health care, including: permanent income, current income, and wealth [ONLINE FILE 1]. The indicator of ‘catastrophic costs due to TB’ is intended to capture where costs associated with TB impose an economic burden that is non-recoverable, beyond typical day-to-day wealth management. Theoretically, permanent income is the best comparator to reach this aim. Measures of permanent income will more appropriately reflect the impact of health costs on the total resources available to the household, thus capturing any potential long-term depletion in financial wellbeing in the household. According to the permanent income hypothesis, permanent income can be captured through consumption expenditure (Friedman, 1957), as consumption stays relatively constant according to one's socio-economic status (Garvy, 1948). A consumption expenditure module should therefore appropriately capture ability to pay for health-related costs.

However, pragmatically most surveys estimating catastrophic costs for specific diseases are conducted with patients attending a health facility, as disease prevalence is often too low to make household surveys efficient. Interviewing at the facility, often as part of clinical trials, introduces substantial time and cost restrictions on the survey. Short-form consumption expenditure questionnaires are not available for many contexts, and the limited time available often prevents full consumption expenditure surveys. The risk of survey fatigue for patients interviewed at a health facility is also much higher and large sample sizes are often not possible (Sweeney et al., 2016). Researchers have therefore opted to take various approaches to estimate ‘capacity to pay’, with the large majority using self-reported current annual income in the denominator of the catastrophic costs equation (Barter et al., 2012) WHO recommendations currently suggest equivalence between current income and annual household expenditure.

Estimates of current income are subject to variation arising from different methods of measurement (diary vs. recall), recall periods, levels of detail in questions soliciting income, and level of respondent (individual vs. household). There is some evidence that each of these factors can lead to bias in income measurement. Bias can manifest in the form of error in reporting (i.e. due to recall error, telescoping, rounding error, cognitive errors, survey fatigue or misreporting), or in the form of non-response (Beegle et al., 2012; Browning et al., 2014; Deaton, 2001; Deaton and Grosh, 1999; Foster and Lound, 1993; Gibson, 2016; Jolliffe, 2001; Moore et al., 2000; Pudney, 2008; Winter 2002, 2004). While it is possible to adjust analysis for partially observed data (i.e. through multiple imputation, mean imputation, or other assumed values) (Brick and Kalton, 1996; Sinharay et al., 2001), income data is susceptible to non-response not at random, making many forms of imputation likely inappropriate. Survey design is key in efforts to limit the amount of missing data.

Another potential solution to the problem of bias in small facility-based surveys is using a proxy for income, either by assuming the national average income for all participants or by using household assets as a proxy for permanent income. Where national survey data exist, it is possible to use principal components analysis or multiple correspondence analysis (MCA) to compute factor weights at the national scale, which can then be applied to asset data for a smaller survey. This approach allows researchers to estimate permanent income without the large expense of conducting a national survey (Gwatkin et al., 2005; McKenzie, 2005; Wagstaff et al., 2007). There are some limits associated with this approach, however; assets are slow-changing and therefore may not capture changes in household economics accurately, particularly for the lowest quintile (Booysen et al., 2008; Harttgen and Vollmer, 2011).

Finally, the issue of income measurement can be avoided entirely by adopting an indicator of financial catastrophe which is not dependent on estimating TB-related costs as a proportion of capacity to pay. Following indications that financial catastrophe is linked with coping strategies (Madan et al., 2015), presence of these strategies could be used as an indicator of catastrophic cost.

## 2 Methods

### 2.1 Study design

We present and compare estimates of catastrophic cost using a range of existing methods to represent household capacity to pay for TB services, in the absence of a full consumption questionnaire. We use data from a patient costing study nested within the TB FastTrack study, a pragmatic, cluster randomised trial with 24 primary healthcare clinics randomised to implement algorithm-guided empirical TB treatment for ambulant HIV-positive adults who had a low CD4 count and were not yet on TB or HIV treatment (Fielding et al., 2015). Patients in the intervention arm were started on TB treatment if indicated by the study algorithm, and ART initiation was promoted either two weeks after the start of TB treatment, or at the earliest opportunity if TB treatment was not indicated; in the control arm, clinic staff initiated TB treatment and/or ART according to routine practice. Patient cost data was collected between June 2014 and March 2015. The patient cost study was not designed to draw any conclusions on the impact of the TB Fast Track intervention on income or cost. Ninety-nine participants were recruited from a pragmatic sub-selection of 17 study facilities in Bojanala Platinum (28 participants), City of Ekurhuleni (9 participants), City of Tshwane (48 participants), and Greater Sekhukhune districts (14 participants). Bojanala Platinum and Greater Sekhukhune are both rural districts, located in North West and Limpopo provinces respectively. City of Tshwane and City of Ekurhurleni are peri-urban districts, both located in Gauteng province. All municipalities had high unemployment rates in 2011, ranging from 24.2% in City of Tshwane to 50.9% in Sekhukhune (Statistics South Africa, 2014a).

Participants were interviewed for this study at their 6-month follow-up trial visit. Questionnaires were adapted from the USAID Tool to Estimate Patient Costs for TB (USAID et al., 2008), and included a series of questions about patient demographics, asset holdings, health care seeking behaviour, costs associated with seeking care, and income [ONLINE FILE 2]. Questionnaires included detailed questions on visits made to a range of providers, including: the trial clinic; other public facilities; general practitioners; hospitals; traditional healers; and pharmacies. Questionnaires were designed to exclude visits that were made solely for research purposes. Data on household size from the survey was unreliable, as data was only available for 49 participants. To maintain consistency in the analysis we used the mean household size by municipality as obtained from Statistics South Africa as a measure of household size rather than individual household estimates.

Data were entered into an Excel spreadsheet, and analysed using a combination of Excel and Stata 14. All cost and income data were inflated using the local inflation rate to reflect prices in October 2015, and then converted to USD using the average conversion rate in October 2015, R 13.08 = 1 USD (XE, n.d.). Participants were interviewed in a private space and all data were anonymised prior to analysis. The trial, including the costing study, was approved by the Research Ethics Committees of the University of Witwatersrand (approval number: R14/49 M111177), the London School of Hygiene and Tropical Medicine (approval number: 6099), and the Provincial Research Committees of Gauteng, North West and Limpopo.

### 2.2 Components of catastrophic cost estimates

We estimated the proportion of households encountering catastrophic costs for each income estimation approach, following WHO definitions of catastrophic costs (World Health Organization, 2017a):

$$\frac{Episode\;direct\;cost+Episode\;indirect\;cost}{Household\;'capacity\;to\;pay'}>THRESHOLD\;VALUE\;\left(\text{\%}\right)$$

Methods of estimation for each of these components are detailed below. For comparison of catastrophic cost incidence across estimation approaches, we use a threshold value of 20% as a base case, but also illustrate the impact of varying threshold on the total proportion of participants encountering catastrophic cost. We also considered the presence of coping strategies as an indicator of catastrophic cost.

#### 2.2.1 Estimation of household ‘capacity to pay’

We estimated the annual household income using four different approaches, described below. We did not attempt to estimate household consumption or expenditure, as at the time of study design there were no validated short-form consumption questionnaires for use in South Africa.

##### 2.2.1.1 Approach #1: self-reported current income (prompted ranges)

On trial enrolment, we asked participants to self-report their monthly household income using a single question with prompted ranges of: less than $62, $62-$104, $104-$208, $208-$415, greater than $415, or not known. Households in each range were assigned the mid-point income for that range (i.e. $31 for those stating income less than $62, $83 for those with income $62-$104, and so forth).

##### 2.2.1.2 Approach #2: self-reported current income (detail)

During the patient costing questionnaire at the 6-month follow-up visit, we asked participants to recall the monthly income of the household with respect to 4 time-points: prior to symptom onset, at trial enrolment, at the start of TB treatment (or HIV treatment if not treated for TB), and at the 6-month follow-up visit. The onset of symptoms was self-identified by participants as the date when they “first felt unwell”. Income was solicited this time with detailed questions surrounding the salary and non-salary income of the participant and that of other household members; questions included monetary income, non-monetary income (e.g. food), grants and remittances. Household income prior to symptom onset was used for the denominator of the catastrophic cost equation.

##### 2.2.1.3 Approach #3: estimated permanent income based on asset scoring

At trial enrolment, we asked participants about a range of assets held by the household and household characteristics, including: a stove, DVD player, motorcar, washing machine, satellite television, computer, radio, television, refrigerator, cell phone, bicycle, and indicators of housing quality (toilet facilities, source of water, wall materials, floor materials, and dwelling type). We used the same asset questions as the National Income Dynamics Survey (NIDS) in South Africa (Leibbrandt et al., 2009), a national panel survey of households. Coding for these questions was mapped to coding for the same questions from the NIDS.

We conducted a multiple correspondence analysis (MCA) on NIDS survey data to estimate weights for each of the above-described assets and characteristics as reported in the most recent round of the survey, conducted in 2015 (Wave 4) (Booysen et al., 2008; Howe et al., 2008). The first dimension explained 78% of variation in the dataset. Weights from the first dimension were applied to the TBFT dataset, and households were classified into five socio-economic quintiles. For each income quintile, mean monthly expenditures were taken from the NIDS dataset and assumed to represent the mean household permanent income for that quintile. This was used as the denominator for catastrophic costs.

##### 2.2.1.4 Approach #4: estimated income based on the average net disposable income

We compared the above income measures against a broad assumption that all households earned the average net adjusted after-tax income in South Africa, as estimated in the OECD Better Life Index (USD 8712 per year) (OECD, 2017). Income estimates were inflated from the 2013 reference year given by OECD to October 2015.

#### 2.2.2 Estimation of direct costs

We estimated direct medical and non-medical costs for the numerator of the catastrophic cost equation. Direct medical and non-medical costs were estimated for the period from the onset of symptoms to the 6-month follow-up visit. If participants had no symptoms, costs were estimated for the three months prior to enrolment to ensure all related care-seeking costs were included. To standardize costs, we assumed a minimum 6-month follow-up period after enrolment for all participants. In cases where participants were interviewed before 6 months, we estimated an average monthly cost and then extrapolated this to six months. The 6-month recall period is longer than typically recommended to estimate costs; as the patient cost study was not designed to provide definitive conclusions on the cost of the TB Fast Track intervention we accepted some risk of bias in order not to interfere with the intervention implementation.

Direct costs were defined as medical and non-medical expenses. Medical expenses included consultation fees and any out-of-pocket payment for medicines and diagnostics paid at any provider type. Direct non-medical expenses included any travel costs of participants and guardians, food costs incurred while in hospital, money spent buying any special foods or dietary supplements due to illness, and any interest incurred on loans taken out to meet the costs of out-of-pocket payments. Direct medical and non-medical costs were determined as the product of the reported expense for the most recent visit to each provider type and the number of visits made to that provider.

#### 2.2.3 Estimation of indirect costs

We estimated indirect costs for the numerator of the catastrophic cost equation using two approaches. First, indirect costs were defined as the opportunity cost of time spent away from the daily productive routine. The number of hours included time spent traveling to health facilities and waiting and consultation time, excluding any extra visits made for research purposes alone. Any time spent by household members caring for the participant or covering household chores usually done by the participant was also included. The total time was multiplied by the estimated household income per person per minute, which was derived from each of the four respective measures of household income estimated as described above using the mean household size by municipality from Statistics South Africa and self-reported working hours per day (approaches #1–4).

Next, we estimated the indirect cost using the self-reported income loss during the period from symptom onset to 6 months after study enrolment. Any gain or loss in income during this time which the participant attributed to illness was considered the total indirect cost. This is labelled as approach #5.

#### 2.2.4 Coping strategies

Finally, we consider use of coping strategies as an indicator of economic catastrophe (approach # 6). Participants were asked about their use of several coping strategies to meet the costs of TB, including asset sales, taking loans, reducing food consumption, and changes in household labour use (e.g. pulling children out of school to work).

### 2.3 Analysis

To facilitate comparison between different income measurement approaches, we began our analysis by dropping all participants for whom a household income was not calculable using any of the income estimation approaches described below due to missing data (n = 33) and conducted a complete case analysis for the remaining participants (n = 66). We tested the reliability between different approaches of income measurement using Cohen's kappa statistic (McHugh, 2012). Finally, we illustrate the resource implications of varying methods using the example of South Africa's temporary disability grant, which is a monthly cash transfer providing income support to all South African citizens who are unable to work due to disease or disability (typically R1010 ($67.23) per month). We estimate the total cost of a years' access to the temporary disability grant ($806.76) for each household identified as encountering catastrophic cost by each approach. This type of grant could protect households from the negative economic ramifications of catastrophic TB costs and reflects the potential cost of reducing catastrophic costs.

## 3 Results

### 3.1 Data and demographics

Ninety-nine people in total participated in the patient costing survey. Of these, we excluded 33 participants (33%) from the full analysis due to missing data for one or both self-reported income questions. Twenty-seven participants responded, “Don't know” to the question “On average, what is your monthly household income: zero or less than R600 ($62), R601-1000 ($62-$104), R1001-2000 ($104–208), R2001-4000 ($208–415), or greater than R4000 ($415)”. When responding to more detailed income questions, two participants were unable to report their own income, and eight were unable to report the income of other household members.

Table 1 shows the demographic data for those participants included in the analysis (n = 66), and for those excluded (n = 33). Most participants included in analyses were female (n = 45), and between the ages of 30 and 44 (n = 47). All participants were of black African ethnic origin, and 89% were educated to grade 8 and above (n = 59). The majority (n = 40) were unmarried. Only 53% (n = 35) of participants reported being employed at the time of symptom onset (or 3 months prior to enrolment if no symptoms); this had dropped to 48% (n = 32) by the time of trial enrolment. Excluded participants were significantly more likely to be unemployed at symptom onset than those included in the analysis.

**Table 1:** Demographic characteristics of study participants, comparing those included vs. excluded in the main analysis.

Variable	Participants included in analysis (n = 66)	Participants excluded due to missing income data (n = 33)	Difference
Female n (%)	45 (68%)	19 (58%)	chi^2^ = 1.08; p = 0.30
Mean age (Std Dev)	37 (8.0)	40.8 (11.9)	t = −1.76; p = 0.08
Black/African n (%)	66 (100%)	33 (100%)	n/a
Grade 8 and above n (%)	59 (89%)	27 (82%)	chi^2^ = 1.11; p = 0.29
Unmarried n (%)	40 (61%)	21 (64%)	chi^2^ = 0.09; p = 0.77
Employed at symptom onset n (%)	35 (53%)	9 (27%)	chi^2^ = 5.91; p = 0.02*
Employed at trial enrolment n (%)	32 (48%)	10 (30%)	chi^2^ = 2.98; p = 0.08
Receiving any government grants n (%)	51 (77%)	24 (73%)	chi^2^ = 0.25; p = 0.62
Receiving disability grant for HIV/TB n (%)	1 (2%)	0 (0%)	chi^2^ = 0.51; p = 0.48
Median CD4 count at last test (IQR)	90 (58)	73 (60)	t = 0.57; p = 0.57
Asset quintile distribution (mapping to national asset index) n (%)	Quintile 1: 3 (5%) Quintile 2: 7 (11%) Quintile 3: 27 (41%) Quintile 4: 18 (27%) Quintile 5: 11 (17%)	Quintile 1: 6 (18%) Quintile 2: 2 (6%) Quintile 3: 8 (24%) Quintile 4: 6 (18%) Quintile 5: 11 (33%)	chi^2^ = 10.23; p = 0.03*
Coping strategies	Coping: 24 (36%) Took loans: 20 (30%) 0–25% interest: 6 (9%) ≥25% interest: 14 (21%) Reduced food: 10 (15%) Sold assets: 2 (3%) Multiple strategies: 8 (12%) No coping: 42 (64%)	Coping: 15 (45%) Took loans: 12 (36%) 0–25% interest: 7 (21%) ≥25% interest: 5 (15%) Reduced food: 0 (0%) Sold assets: 4 (12%) Multiple strategies: 2 (6%) No coping: 18 (55%)	chi^2^ = 0.76; p = 0.38

IQR interquartile range.

Many households undertook coping strategies to meet the costs of illness. Several households reported reducing food consumption (n = 10), selling assets (n = 2) or taking out loans (n = 20), but no households reported taking children out of school to work. Some participants who were excluded from the analysis due to missing income data sold assets (n = 4) or took loans (n = 12) to meet costs related to illness. The median CD4 count reported at enrolment was 90.

### 3.2 Total direct costs

Mean direct medical costs for all providers per episode were $23 and mean costs for travel and food during this period were $37 (Table 2). Sixteen participants visited general practitioners at least once at an average cost of $24 per visit, and seven participants were hospitalized at least once. No patients in this cohort received daily clinic-based directly observed treatment (DOT). Eight participants visited a traditional healer at least once, with consultation fees per visit ranging from $5 to $97 per visit. Direct non-medical costs were highest for participants' main clinic – this reflects travel and food costs for participants and their guardians during the many visits to these facilities. Supplementary file 3 shows the visit and direct cost data for excluded participants [ONLINE FILE 3].

**Table 2:** Mean number visits, direct costs, and time spent seeking care from start of illness to 6-month trial visit (n = 66).

Facility type	Mean total number visits	Mean total direct medical cost	Mean total direct non-medical cost	Mean total hours care-seeking
Main clinic	12.98	$0.00	$27.32	70.01
Other clinic	0.12	$0.00	$0.31	1.03
Pharmacy	1.44	$4.60	$0.86	1.51
General practitioner	0.35	$7.56	$0.86	1.27
Hospital-inpatient	0.12	$0.80	$4.49	8.25
Traditional healer	0.21	$8.95	$0.69	1.56
Specialist	0.57	$0.57	$1.19	1.97
Radiologist	0.00	$0.00	$0.88	1.02
DOT	0.00	$0.00	$0.00	0.00
**Total**	**15.80**	**$22.48**	**$36.60**	**86.62**

All costs in 2015 USD.

### 3.3 Total resources available to the household

Table 3 shows the mean and standard deviation of the estimated resources available to the household, and the number of participants falling below the nationally defined lower-bound poverty line of $43 per person/month for each of the four income estimation approaches (Statistics South Africa, 2014b).

**Table 3:** Monthly household income estimates using different approaches (n = 66).

Income estimation approach	Households below poverty line	Mean monthly income per household	Median monthly income per household	Standard Deviation
Approach#1: current income (prompted ranges)	40	$241.70	$156.00	221.03
Approach#2: current income (detailed)	33	$317.71	$221.80	340.88
Approach#3: permanent income (MCA)	2	$497.33	$339.23	289.92
Approach#4: national mean income	0	$760.70	$760.70	–

All income in 2015 USD.

The two methods with the highest correlation coefficient (0.373; p = 0.002) were approach #1 and approach #2. The mean monthly income per household measured using prompted ranges (approach #1) was $242 (median $156), and the mean income reported in response to detailed questions (approach #2) was $317 (median $222).

Weights for assets and household characteristics from the NIDS MCA exercise are listed in Supplementary file 4 [ONLINE FILE 4]. All durable assets had positive factor loading scores while indicators of poor housing had negative loading scores. Durable asset ownership was moderately correlated with permanent income in the NIDS dataset (r = 0.40; p < 0.00). More participants in the TBFT dataset reported ownership of some durable assets and high-quality housing characteristics, placing more participants in higher income quintiles than lower quintiles. Permanent income estimated using the MCA approach (approach #3; mean $497) was significantly higher than self-reported current income (approaches #1 and #2) (p < 0.002).

The highest mean income was estimated using approach #4 (mean $761); income estimates for approach #4 were also significantly higher than those for approaches #1 and #2 (p < 0.00). Depending on the approach taken to estimate income, as few as zero or as many as 40 of the 66 (61%) households were estimated to fall below the poverty line.

### 3.4 Total indirect costs

Indirect costs were a function of income and followed the same pattern as that of income. The highest indirect costs were estimated using approach #4, and the lowest indirect costs were estimated using approach #1. Depending on the income estimation approach taken, mean indirect costs for the episode varied from a mean of $33 to $113. Differing approaches in income estimation therefore had wide ranging impact on cost drivers overall. Indirect costs account for 64% of total cost when using approach #4 for income estimation, and 34% of total cost when using approach #1 (Table 4). Self-reported income loss (approach #5) was roughly double that of time loss valued in terms of current income (approaches #1 and #2), and had a much larger standard deviation than any other approach – this was due to a few participants reporting substantial income loss as a result of job loss due to illness, and a few reporting substantial income gains (e.g. in grants or remittances) as a result of their illness.

**Table 4:** Indirect costs for all estimation approaches from start of illness to 6-month trial visit (n = 66).

Indirect cost estimation approach	Mean indirect cost	Standard deviation	Indirect cost as % total cost
Approach#1: current income (prompted ranges)	$33.33	53.16	34%
Approach#2: current income (detailed)	$43.55	53.80	41%
Approach#3: permanent income (MCA)	$74.75	77.62	54%
Approach#4: national mean income	$113.77	95.96	64%
Approach#5: self-reported income loss	$85.85	744.08	57%

All costs in 2015 USD.

### 3.5 Catastrophic costs

**Fig. 1:**
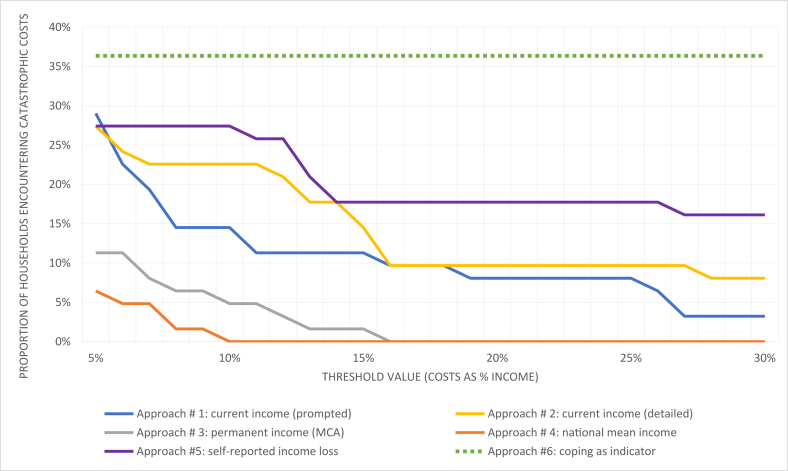
Prevalence of catastrophic cost, by income estimation approach and threshold value.

Fig. 1 illustrates the proportion of participants in the study encountering catastrophic costs across a range of thresholds, by approach. Across thresholds and particularly at lower thresholds, the choice of income measure lead to very large differences in the proportion of catastrophic cost. Approaches #3 and #4 had the fewest participants encountering catastrophic costs, dropping to zero at thresholds above 10%.

Table 5 presents the estimated prevalence of catastrophic cost for each of the six estimation approaches, and the potential cost of providing a disability grant to those encountering catastrophic costs. Under the national average income assumption, zero participants encountered cost over the 20% threshold. Using self-reported income data, six participants (9% of those included in analysis) encountered catastrophic costs. Using coping as an alternative indicator of catastrophic costs, 24 (36%) encountered catastrophic costs. There was minimal agreement between the five income measurement approaches in identification of catastrophic cost (Kappa = 0.2711, p < 0.000). Participants who reduced food consumption to meet costs were largely not classified as encountering catastrophic costs under approaches #1–4, however approach #5 reflected catastrophic costs for some of these participants.

**Table 5:** Policy impact of catastrophic cost estimates.

	Number participants with catastrophic cost	Total cost of providing one year disability grant to all households with catastrophic cost
	(total n = 66)	
Approach#1: current income (prompted ranges)	6 (9%)	$7997.23
Approach#2: current income (detailed)	6 (9%)	$7997.23
Approach#3: permanent income (MCA)	0 (%)	$0.00
Approach#4: national mean income	0 (%)	$0.00
Approach#5: self-reported income loss	11 (17%)	$14,661.58
Approach#6: coping strategies	24 (36%)	$31,988.90

Catastrophic threshold for Approaches #1-#5: 20%.

All costs in 2015 USD.

Of all patients interviewed, only one was in receipt of a disability grant related to their HIV/TB status. If all those undertaking coping strategies were assumed to encounter catastrophic costs, the cost of providing disability grants to those people would be $31,988. In contrast, if the national average income is used to estimate income, zero participants would be found to encounter catastrophic costs and there would be zero cost to providing disability grants.

## 4 Discussion

This paper illustrates the uncertainty around measuring income accurately when estimating disease-specific catastrophic costs. The gold standard for estimating permanent income is through a consumption expenditure questionnaire. In this setting, as in many real-world situations, it was not possible to conduct such a questionnaire due to time limitations and the lack of a validated short-form questionnaire. In the absence of such a gold standard, we illustrate the implications of alternative approaches. The four income measurement methods we employed gave substantially different estimates of the frequency of catastrophic costs with vastly different policy implications; different approaches in estimating income amongst the same population resulted in estimates varying from 0 to 36% of respondents encountering catastrophic costs.

It is clear from our results that all potential alternatives presented are problematic in some way. Self-reported current income, as estimated through approaches #1 and #2, is a poor proxy for permanent income. In addition, these data were the most difficult to collect amongst the five approaches. We lost 33 participants from our analysis due to missing data for one or both self-reported income estimation approaches. We lost a disproportionate number of participants who were unemployed and who adopted certain coping strategies, including selling assets and taking loans to meet the costs of TB, potentially biasing our results to reduce the estimated prevalence of catastrophic costs. This loss of data is not unusual for this kind of survey. The practical difficulties of collecting reliable income data are widely acknowledged (Deaton and Zaidi, 2002; Filmer and Pritchett, 2001), and it is often particularly difficult for participants to estimate income outside the purview of the survey respondent, which is critical for estimation of household income. It is crucial when estimating catastrophic costs to ensure that the analysis is not biased against capturing those who encounter serious difficulty in meeting the costs of illness.

Income quintiles were estimable using the MCA approach (approach #3) for all 99 participants; however, there are several potential limitations with this approach as illustrated in this paper. Our sample had relatively high levels of durable asset ownership, placing many participants in the upper two quintiles and resulting in only two households being defined as below the poverty line using approach #3. This indicates that approach #3 may have substantially overestimated household socioeconomic position, as consistent evidence indicates that both TB and HIV are most prevalent among lower income quintiles (Lönnroth et al., 2009; Steinert et al., 2017; Wabiri and Taffa, 2013). This approach also assumes that expenditure patterns of TB-affected households are similar to the national average, which is unlikely to be the case. Although theoretically promising, we must therefore draw the conclusion that asset indices are likely a poor proxy for consumption expenditure in the South African setting. This is consistent with indications that asset indices are poor proxies for consumption expenditure across a range of settings (Howe et al., 2009). Asset indices also may not be the best option available to researchers - asset questionnaires can be very lengthy in themselves, and mapping to a national dataset is not always possible. Researchers looking to use asset mapping to proxy permanent income should first check whether there is a national dataset that can be mapped to assets in a facility survey and whether there is a high correlation with permanent income in their setting. Increasing the number and range of indicators may help to improve agreement.

As expected, the use of a mean national income in the denominator (approach #4) was highly problematic. The approach likely substantially overestimated household socioeconomic position and provided no real sense of the relative impact of TB costs across socio-economic quintiles. This approach does not achieve the aim of the indicator of ‘catastrophic costs due to TB’ and adds no value to a blunt estimate of total costs due to TB.

Given the limitations of methods to estimate catastrophe quantitatively in absence of consumption expenditure, we also explored the use of alternative measures such as adoption of coping behaviours (approach #6) as an indicator of catastrophic costs. Unlike some quantitative measures explored, this information was easily collected for all households. Coping strategies may be a good indication of long-term financial hardship in the context of health-related costs. Health shocks are often costlier than other types of shocks, and households are often less able to recover following a health shock as compared with agricultural, natural, or legal shocks (Dhanaraj, 2016; Heltberg and Lund, 2009). This is especially the case when illness is repeated, or in the case of chronic illness, such as HIV and TB (Gertler and Gruber, 2002; Kenjiro, 2005; Wagstaff and Lindelow, 2014). Our data indicates that several households reduced food consumption to meet health-related costs, which can lead to under-nourishment, increase susceptibility to infectious disease, reduce quality of life, and damage long-term productivity. Many households also took loans at high interest, potentially leading to unmanageable debt. Most of these participants were not classified as encountering catastrophic cost using approaches #1-#5, despite the potential long-term effects of these coping strategies.

However, as noted by Collins et al. many households living near the poverty line frequently take loans and sell assets in their day-to-day management of resources (Collins et al., 2009). The high frequency of coping strategies employed by all households in the sample could reflect households using all the resources available to them in a dynamic process of managing assets to raise funds to pay for illness-related expenses, rather than an act of desperation. Indeed, the greatest long-term difficulty might be encountered by those households which do not have assets to sell, are not creditworthy or otherwise unable to take out loans, or cannot further reduce food intake. Further research linking use of coping strategies to long-term economic outcomes within households would help to better identify the potential usefulness of this metric.

This study was designed to illustrate and explore the challenges around measuring income at the facility level and has some clear limitations. Our sample size was small and was further limited by missing income data which led us to drop 33 participants from the analysis. While these limitations do not impact the validity of our observations about missing data and internal comparisons, results should not be taken as evidence surrounding catastrophic costs for people with TB/HIV in South Africa or any conclusions on the TB Fast Track trial. We did not include direct costs of childcare in our questionnaire, potentially underestimating direct costs. We used the average household size by municipality to estimate income per person, thereby introducing some uncertainty into our estimates. We did not collect information on which assets were sold, and therefore are unclear how asset sales may have impacted household placement in the asset index. We also were not able to compare against a gold standard of household income measurement such as a household consumption survey, and thus have no way to test the degree to which bias may have affected our findings. However, this paper highlights the extent of uncertainty around these measures and the need for greater clarity on the most appropriate measure of household resources for estimating catastrophic costs.

We did not explore here the approach of using consumption-based measures for patients attending health facilities, yet these may also be considered. Short-form consumption questionnaires have been successfully used in surveys in the past (Wagstaff and Lindelow, 2014), although short-form questionnaires have not yet been validated for many settings. Further development and validation of short-form consumption questionnaires would greatly improve ability to measure permanent income in a facility-based setting. There should also be further investigation into whether short-form consumption questionnaires are needed at all, or whether a full consumption module might be preferable given the potentially high expected value of information associated with these surveys. In a recent implementation of the Living Standards Measurement Survey, the full consumption module took an average of only 25 min (Browning et al., 2014); it may therefore be feasible to implement full consumption modules in facility-based surveys if other questions can be reduced.

There is growing concern to provide social protection to those facing catastrophic costs due to TB (Boccia et al., 2011; Siroka et al., 2016). Improved social protection could help to mitigate long-term costs through improved TB and other health outcomes, reduced periods of time off work, and increased productivity. As demonstrated in this paper, the additional costs faced by countries which will be liable for social protection for those facing catastrophic costs are potentially substantial. In the absence of a gold standard to identify those needing social protection, the substantial uncertainty identified in this paper opens the possibility of gaming, or choosing a particular method for measuring income to minimize the frequency of catastrophic costs, for example to appeal to funders or to minimize the cost of social protection. It is also possible that some countries will be unfairly judged as performing worse than others when the estimation method is simply different.

Using existing data, this paper shows the potential implications of different measures of household resources in the denominator of the catastrophic cost equation. Further concerted research is needed to come to an acceptable recommendation for measurement of TB-specific catastrophic costs, and in the meanwhile countries and economic evaluators should use a range of approaches. New guidelines developed by the Global Health Cost Consortium (GHCC) highlight the importance of stating potential sources of bias clearly in cost estimates for health interventions (Vassall et al., 2017) for use in economic evaluation and more generally. We suggest that methods for estimating income, and potential sources of bias arising from these methods are clearly explained and discussed to facilitate interpretation. Our findings confirm the recommendation by the WHO Task Force to use multiple methods for income estimation, and stress that different approaches should not be used as substitutes for one another until these measures can be directly compared against consumption modules. Further research is needed to evaluate the benefits and drawbacks of these different approaches, and to empirically validate rapid estimation methods which can be used in a facility setting.

## Appendix A Supplementary data

SUPPLEMENTARY FILE 1_Glossary SUPPLEMENTARY FILE 1_Glossary

SUPPLEMENTARY FILE 2_Data collection questionnaire SUPPLEMENTARY FILE 2_Data collection questionnaire

SUPPLEMENTARY FILE 3_Detailed costs for included and excluded participants SUPPLEMENTARY FILE 3_Detailed costs for included and excluded participants

SUPPLEMENTARY FILE 4_MCA results SUPPLEMENTARY FILE 4_MCA results

SSM-D-17-03499_DataProfile SSM-D-17-03499_DataProfile
