# Supplementary material for: Measuring income for catastrophic cost estimates: Limitations and policy implications of current approaches
Source: Soc Sci Med. 2018 Oct;215:7–15. doi: 10.1016/j.socscimed.2018.08.041 (PMC6171470; doi:10.1016/j.socscimed.2018.08.041)
Supplement: SUPPLEMENTARY FILE 1_Glossary [file mmc1.docx]

# Appendix 2: Glossary

## Current income:

The amount earned by a person or household at any one given time. This can include: cash and non-cash earnings from productive activities; rental income from the supply of land, capital, or other assets; businesses; current transfers from government or non-government agencies or other households, and/or investments (O’Donnell and Wagstaff, 2008; Wai-Poi et al., 2008). Current income tends to be lumpy and can be seasonal or dependent on the local labour market. Current income does not reflect important assets (such as savings) that can be drawn upon to finance health care without affecting economic wellbeing within the household (Flores et al., 2008; O’Donnell and Wagstaff, 2008).

## Permanent income:

The long-term average income expectations of a person and/or household (Friedman, 1957; Hall, 1978; Meghir, 2004). According to the permanent income hypothesis, households ‘smooth’ their income by spending less in times of high income and borrowing or drawing on savings in times of low income.

## Consumption:

The resources actually consumed by a household, including: food items; non-food items; consumer durables; and housing. Consumption includes purchased items and items produced at home (eg. food that is grown at home) (Deaton and Grosh, 1999; O’Donnell et al., 2007).

## Consumption expenditure:

Money spent on goods and services consumed by the household (Howe et al., 2012). According to the permanent income hypothesis, consumption expenditure is a more accurate reflection of household living standards than current income (Friedman, 1957), as consumption stays relatively constant according to one’s socio-economic status (Garvy, 1948).

## Coping strategies:

A form of consumption smoothing, which allows households to manage the costs of financial shocks. For example, when faced with a minor illness, a household will typically cope by drawing upon its available resources to smooth the financial shock of illness, reducing the impact on non-health spending and avoiding impoverishment (Wagstaff, 2008). There are a variety of methods that households can employ to cope with health shocks, either by generating cash to meet out of pocket payments, or through rearranging human or social capital to cope with indirect costs. Common coping strategies include: mobilizing savings, deferring expenditure, selling assets, taking loans, income diversification, taking on additional labour, or relying on gifts/mutual support networks (Bharadwaj, 2014; Goldman and Smith, 2001; Sauerborn et al., 1996). In addition, in many subsistence-based economies family members will fill in for a sick person during the planting season in agriculture (Su et al., 2007). Coping strategies can help households manage transitory shocks (Wagstaff, 2008), however consumption smoothing or other risk management mechanisms can break down under repeated shocks or long-term shocks (Alderman, 1996; Dercon, 2002). When households are unable to manage shocks through coping strategies, they might take more desperate adaptive measures - such as depleting productive assets, removing children from school, reducing consumption, sex work, begging, and crime - which can lead to an increased cycle of vulnerability (Alwang et al., 2001; Ilboudo et al., 2013; Sauerborn et al., 1996).

## Direct costs:

Direct costs include any direct expenditures associated with illness, or with accessing care (Cooper and Rice, 1976; McIntyre et al., 2006; Rice, 1967). This includes direct medical costs (for example money paid for medicines, diagnostics, consultation fees, or informal payments made to health workers) and direct non-medical costs (for example transport costs to attend a health facility, accommodation costs whilst seeking care, costs of any special food or supplements taken as a result of illness, or costs of childcare).

## Indirect costs:

Indirect costs refer both to the opportunity costs of time spent by the patient and household members in seeking care (eg. travel time, waiting time, and time in consultations), time spent by household members who provide informal care for the patient, and time spent unproductive as a result of illness (McIntyre et al., 2006).

## Productivity costs:

Productivity costs are a subset of indirect costs, and refer to the time spent unproductive as a result of illness. Productivity costs are incurred in the form of absenteeism and presenteeism

## Wealth:

The aggregate value of all household assets and holdings (Wai-Poi et al., 2008). Households use a variety of assets as wealth stores, and can invest in or draw on these investments in order to mitigate income shocks (Alwang et al., 2001; Scoones, 1998; Sen, 1981). The specific assets available to a household have been classified in the literature differently depending on the framework (Bebbington, 1999; Moser, 1998; Scoones, 1998; Wallman and Baker, 1996), but often include: human capital (skills, good health and ability to labour, ability to pursue livelihoods), natural capital (land, water, environmental resources), physical capital (basic infrastructure of housing, water, transport, electricity), financial capital (savings, credit, pensions etc. which produce livelihood options), and social capital **(**networks, associations, institutions on which people can draw) (Scoones, 1998). Households can store, accumulate, exchange, deplete, or put these assets to work in their day to day management of risk and generation of income (Rakodi, 1999).

# References

Alderman, H., 1996. Saving and economic shocks in rural Pakistan. J. Dev. Econ. 51, 343–365. https://doi.org/10.1016/S0304-3878(96)00419-1

Alwang, J., Siegel, P.B., Jørgensen, S.L., Tech, V., 2001. Vulnerability : A View From Different Disciplines. Soc. Prot. Discuss. Pap. . World Bank 46.

Bebbington, A., 1999. Capitals and Capabilities: A Framework for Analyzing Peasant Viability, Rural Livelihoods and Poverty. World Dev. 27, 2021–2044. https://doi.org/10.1016/S0305-750X(99)00104-7

Bharadwaj, A., 2014. Is Poverty the Mother of Crime? Empirical Evidence of the Impact of Socio-Economic Factors on crime in India. Atl. Rev. Econ. 1, 1–40.

Cooper, B.S., Rice, D.P., 1976. The economic cost of illness revisited. Soc. Secur. Bull. 39, 21–36.

Deaton, A., Grosh, M. (Eds.), 1999. Designing Household Survey Questionnaires for Developing Countries: Lessons from 15 years of the Living Standards Measurement Study. The World Bank, Washington, D.C.

Dercon, S., 2002. Income Risk, Coping Strategies, and Safety Nets (No. 2002/22), WIDER Discussion Papers. Helsinki, Finland. https://doi.org/10.1093/wbro/17.2.141

Flores, G., Krishnakumar, J., O’Donnell, O., Van Doorslaer, E., 2008. Coping with health-care costs: Implications for the measurement of catastrophic expenditures and poverty. Health Econ. 17, 1393–1412. https://doi.org/10.1002/hec.1338

Friedman, M., 1957. The permanent income hypothesis, in: A Theory of the Consumption Function. Princeton University Press, Princeton, pp. 20–37. https://doi.org/10.1016/S0304-3932(98)00063-4

Garvy, G., 1948. The Role of Dissaving in Economic Analysis. J. Polit. Econ. 56, 416–427. https://doi.org/10.1086/256725

Goldman, D.P., Smith, J.P., 2001. Methodological biases in estimating the burden of out-of-pocket expenses. Heal. Serv Res 35, 1357–1365.

Hall, R.E., 1978. Stochastic Implications of the Life Cycle-Permanent Income Hypothesis: Theory and Evidence. J. Polit. Econ. 86, 971–987. https://doi.org/10.1086/260724

Howe, L.D., Galobardes, B., Matijasevich, A., Gordon, D., Johnston, D., Onwujekwe, O., Patel, R., Webb, E.A., Lawlor, D.A., Hargreaves, J.R., 2012. Measuring socio-economic position for epidemiological studies in low-and middle-income countries: A methods of measurement in epidemiology paper. Int. J. Epidemiol. 41, 871–886. https://doi.org/10.1093/ije/dys037

Ilboudo, P., Russell, S., D’Exelle, B., 2013. The Long Term Economic Impact of Severe Obstetric Complications for Women and Their Children in BurkinaFaso. PLoS One 8.

McIntyre, D., Thiede, M., Dahlgren, G., Whitehead, M., 2006. What are the economic consequences for households of illness and of paying for health care in low- and middle-income country contexts? Soc. Sci. Med. https://doi.org/10.1016/j.socscimed.2005.07.001

Meghir, C., 2004. A retrospective on Friedman’s theory of permanent income. Econ. J. 114. https://doi.org/10.1111/j.1468-0297.2004.00223.x

Moser, C., 1998. The asset vulnerability framework: Reassessing urban poverty reduction strategies. World Dev. 26, 1–19. https://doi.org/10.1016/S0305-750X(97)10015-8

O’Donnell, E., Wagstaff, A., Lindelow, M., van Doorslaer, O., 2007. Measuring Living Standards: Household Consumption and Wealth Indices, in: Analyzing Health Equity Using Household Survey Data. World Bank Institute Development Studies, pp. 1–11.

O’Donnell, O.A., Wagstaff, A., 2008. Analyzing health equity using household survey data: a guide to techniques and their implementation. World Bank Publications.

Rakodi, C., 1999. A Capital Assets Framework for Analysing Household Livelihood Strategies: Implications for Policy. Dev. Policy Rev. 17, 315–342. https://doi.org/10.1111/1467-7679.00090

Rice, D.P., 1967. Estimating the cost of illness. Am. J. Public Health 57, 424–40. https://doi.org/10.2105/AJPH.57.3.424

Sauerborn, R., Adams, A., Hien, M., 1996. Household strategies to cope with the economic costs of illness. Soc. Sci. Med. 43, 291–301. https://doi.org/10.1016/0277-9536(95)00375-4

Scoones, I., 1998. Sustainable rural livelihoods: a framework for analysis. IDS Work. Pap.

Sen, A., 1981. Poverty and famines: an essay on entitlement and deprivation. Oxford University Press, Oxford.

Su, T.T., Sanon, M., Flessa, S., 2007. Assessment of indirect cost-of-illness in a subsistence farming society by using different valuation methods. Health Policy (New. York). 83, 353–362. https://doi.org/10.1016/j.healthpol.2007.02.005

Wagstaff, A., 2008. Measuring financial protection in health (No. 4554), Policy Research Working Paper Series. Washington, D.C.

Wai-Poi, M., Spilerman, S., Florencia Torche, 2008. Economic well-being: concepts and measurement with asset data (No. 2008–20), NYU Population Centre Working Paper Series. New York.

Wallman, S., Baker, M., 1996. Which resources pay for treatment? A model for estimating the informal economy of health. Soc. Sci. Med. 42, 671–679. https://doi.org/10.1016/0277-9536(95)00412-2
