# Supplementary material for: Measuring income for catastrophic cost estimates: Limitations and policy implications of current approaches
Source: Soc Sci Med. 2018 Oct;215:7–15. doi: 10.1016/j.socscimed.2018.08.041 (PMC6171470; doi:10.1016/j.socscimed.2018.08.041)
Supplement: SUPPLEMENTARY FILE 2_Data collection questionnaire [file mmc2.pdf]

Patient trial ID number: ______________
Clinic Code ______________
Intervention or control arm? (please tick only 1) Intervention [ ] Control [ ]
Have you received treatment for TB? Yes [ ] No [ ]

I would like to now create a simple visual **timeline of your illness with you**:

Period 1: "This is the period from when you first felt unwell until you entered the trial (up to a maximum of 6 months). If you did not feel unwell before entering the trial, this is the period in the 3 months before you entered the trial"

Period 2: "This is a period only for people who were investigated for TB. It is the period from your enrolling in the trial until you were either started on TB treatment or until the decision was made that you did not have TB ." There is no period 2 if you were a. in the intervention arm of the study or b. if you were never investigated for TB.

Period 3: "If you have started TB treatment, Period 3 is the time from when you started TB treatment until to 6 months into the study (ie. now)". If you have not received TB treatment, this is the period from when you enrolled in the study until 6 months into the study, ie. now".

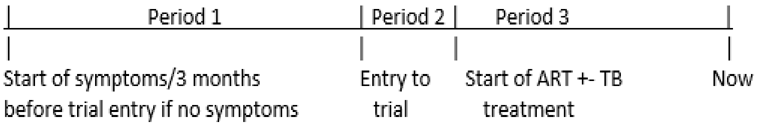

**Period 1 start date (approx to mid/early/late month if patient knows)**	_______ 201_		
**Period 2 start date (entry to trial)**	__/___/201_		
**Period 3 start date (start of TB meds or start of ART if TB not present)**	__/___/201_		
Start date of taking TB meds	__/___/201_		
Start date of taking ART	__/___/201_		
**Today's date (end of Period 3)**	__/___/201_		

**WARMUP**			
1.0	Why did you initially seek medical care ?	[ ] I felt unwell<br>[ ] I went to a medical provider for another reason but was not feeling unwell<br>[ ] I wanted a routine HIV test<br>[ ] Don't know	if "I felt unwell" then skip to 2. otherwise skip t"healthcare expenditure period 1"
	Other____<br>____		
2.0	Had you tested positive for HIV **6 or more months before starting on this trial?** (please tick only one response)	[ ] Yes<br>[ ] No<br>[ ] Don't know	
**HEALTHCARE EXPENDITURE PERIOD 1**			
**Non-specialist clinic visits - Show the participant period 1 on the timeline you have drawn together**			
1.0	This whole question concerns Period 1. In Period 1, how many times did you visit a nonspecialist medical clinic, including this trial clinic, and including the clinic's emergency room (not including a GP (private doctor))?	HIV test:<br>_____times OR [ ] don't know<br>Compliance appointments _____times OR [ ] don't know<br>Emergency room<br>_____times OR [ ] don't know<br>Other clinic appointments<br>_____times OR [ ] don't know	Include any adherence appointments and HIV tests. If "no times", skip to 2.0. do not include enrolment visit. If had 2 clincs on the same day please enter as "other". CD4 test = "other"
1.1	How long, on average, did you spend **waiting for, and in**, an appointment you had at these clinics in period 1?	HIV test:<br>___:___ hours OR [ ] don't know<br>Compliance appointments<br>___:___ hours OR [ ] don't know<br>Emergency room<br>___:___ hours OR [ ] don't know<br>Other clinic appointments<br>___:___ hours OR [ ] don't know	please fill in "0:00" if patient did not have specified visit type
1.2	What is the method of transport you use most often to get to these clinic appointments in period 1?	car [ ]<br>bicycle [ ]<br>motorbike/scooter [ ]<br>by foot [ ]<br>by public transport [ ]<br>privately hired taxi [ ]	If public transport or hired taxi, proceed to 1.7. Other answers - skip to 1.8
1.3	If you took public transport or hired a taxi, how much did you spend, on average, for a journey to and from this/these clinic(s)	__________ Rand OR<br>[ ] nothing OR<br>[ ] don't know	
1.4	How long does the journey take to go to and from your clinic from home?	____:____ hours OR<br>[ ] don't know	

1.5	On average, how much did you need to spend on food on a single one of these clinic visits in period 1?	__________ Rand OR<br>[ ] nothing OR<br>[ ] don't know	
1.6	On visits when you were accompanied, how many adults, on average, accompanied you to these visits? And how many of the visits in this period were they there for?	____ adults for _____visits<br>[ ] no one accompanied me OR<br>[ ] don't know	If "no one accompanied me" then skip to 2.0
1.7	On average, how long would another adult spend accompanying you to on an average clinic visit in period 1? Include **waiting time, appointment time and time taken for the return journey**	____:____ hours OR<br>[ ] no one accompanied me OR<br>[ ] don't know	
1.8	How much, per visit, has been spent on **accommodation, food and transport** that the main adult accompanying you needed because of these visits to these clinics in Period 1?	__________ Rand OR<br>[ ] nothing OR<br>[ ] don't know	
**Hospital/Hospice Admissions**			
2.0	This whole question concerns **Period 1.** In period 1, how many times, if any, were you admitted to **hospital or a hospice?**	__________times OR<br>[ ] no times OR<br>[ ] don't know	If "no times", skip to 3.0
2.1	How long was/were your admissions to hospital or a hospice, in period 1?	Visit 1: _________ days<br>Visit 2: _________days<br>Visit 3:_________days<br>[ ] don't know	
2.2	How much did you pay in fixed charges or fees on your last hospital or a hospice admission in period 1, including "opening a file"?	__________ Rand OR<br>[ ] nothing OR<br>[ ] can't remember	
2.3	What is the method of transport you used to get to your last hospital or hospice admission in period 1?	car [ ]<br>bicycle [ ]<br>motorbike/scooter [ ]<br>by foot [ ]<br>by public transport [ ]<br>privately hired taxi [ ]<br>ambulance [ ]	If **public transport**, or **ambulance** proceed to 2.8. other answers - skip to 2.9
2.4	If you took public transport or hired a taxi or an ambulance, how much did you spend for a journey to and from hospital or the hospice?	__________ Rand OR<br>[ ] nothing OR<br>[ ] don't know	

2.5	How long does the journey take to go to and from hospital or the hospice from home?	____:____ hours OR<br>[ ] don't know	
2.6	On your hospital or hospice admission in period 1, approximately how many days did you have one or more visitors present?	_________ days OR<br>[ ] no-one accompanied me<br>[ ] don't know	If "no one accompanied me" then skip to 3.0
2.7	On days when you had visitors, how many visitors did you have, on average?	_________ visitors OR<br>[ ] don't know	
2.8	On average, on days when you had a visitor, how many hours did your visitor spend visiting you and on the journey time for the return journey to and from hospital or hospice on a single visit in period 1?	____:____ hours OR<br>[ ] don't know	
2.9	How much, if anything, was spent on accommodation that that person needed because of your last hospital or hospice admission in period 1?	__________ Rand OR<br>[ ] nothing OR<br>[ ] don't know	
2.10	How much, per visit, was spent on food and transport that your visitor needed to eat while accompanying you during this admission to hospital or a hospice in period 1?	__________ Rand OR<br>[ ] nothing OR<br>[ ] don't know	
**GP (private doctor)**			
3.0	This question concerns Period 1. In period 1, how many visits did you make to your GP (private doctor) ?	__________times OR<br>[ ] no times OR<br>[ ] don't know	If "no times", skip to 5.0
3.1	How long, on average, did you spend waiting for, and in, an appointment you had at your GP (private doctor) in period 1?	____:____ hours OR<br>[ ] don't know	
3.2	How much have you paid in total for all Xray/radiology tests that your GP (private doctor) requested for you in total across all your visits to him/her in period 1?	__________ Rand OR<br>[ ] nothing OR<br>[ ] don't know	
3.3	How much have you paid for tests from your GP (private doctor) in total across all your visits that were not X rays/radiology in period 1?	__________ Rand OR<br>[ ] nothing OR<br>[ ] don't know	
3.4	How much have you paid in fixed charges or fees in total in visits to your GP (private doctor) in period 1?	__________ Rand OR<br>[ ] nothing OR<br>[ ] don't know	

3.5	What is the method of transport you use most often to get to these GP (private doctor) appointments in period 1?	car [ ]<br>bicycle [ ]<br>motorbike/scooter [ ]<br>by foot [ ]<br>by public transport [ ]<br>privately hired taxi [ ]	If public transport or hired taxi, proceed to 4.6. Other answers - skip to 4.7
3.6	If you took public transport or hired a taxi, how much did you spend, on average, for a journey to and from your GP (private doctor) in period 1?	__________ Rand OR<br>[ ] nothing OR<br>[ ] don't know	
3.7	How long does the journey take to go to and from your GP (private doctor) from home?	____:____ hours OR<br>[ ] don't know	
3.8	On average, how much did you need to spend on food on a single one of these GP (private doctor) visits in period 1?	__________ Rand OR<br>[ ] nothing OR<br>[ ] don't know	
3.9	On visits when you were accompanied, how many adults, on average, accompanied you to these visits? And how many of the visits in this period were they there for?	____ adults for _____visits<br>[ ] no one accompanied me OR<br>[ ] don't know	If no one accompnaied patient, please insert "0" and skip to next section
3.10	How long would another adult spend accompanying you to on an average visit to the private doctor in GP (period 1)? Include **waiting time, appointment time and time taken for the return journey**	____:____ hours OR<br>[ ] no one accompanied me OR<br>[ ] don't know	
3.11	How much, per visit, has been spent on accommodation, food and transport that the main adult accompanying you needed because of these visits to the GP (private doctor) in Period 1?	__________ Rand OR<br>[ ] nothing OR<br>[ ] don't know	
**Pharmacies (not in a hospital or clinic, not a traditional healer)**			
4.0	This question is about Period 1. In period 1, how many times did you go to a pharmacy because of your health?	__________times OR<br>[ ] no times OR<br>[ ] don't know	
4.1	In period 1, how many times did someone else go to a pharmacy because of your health?	__________times OR<br>[ ] no times OR<br>[ ] don't know	**If "no times" is answer to to 5.0 and 5.1, skip to 6.0**

4.2	What is the method of transport you (or the person going to pharmacy for you) used most often to get to a pharmacy in period 1?	car [ ]<br>bicycle [ ]<br>motorbike/scooter [ ]<br>by foot [ ]<br>by public transport [ ] privately hired taxi [ ]	If public transport or hired taxi, proceed to 5.3 Other answers - skip to 5.4
4.3	How much would you spend on drugs from the pharmacy in total in period 1?	__________ Rand OR<br>[ ] nothing OR<br>[ ] don't know	
4.4	If you (or the person going to pharmacy for you) took public transport or hired a taxi or hired a taxi, how much did you spend, on average, for a journey to and from this pharmacy?	__________ Rand OR<br>[ ] nothing OR<br>[ ] don't know	
4.5	How long does the journey take to go to and from your pharmacy from home, including waiting time for the drugs to be made ready?	____:____ hours OR<br>[ ] don't know	
4.6	On average, how much did you or the person going for you need to spend on food on a single one of these pharmacy visits in period 1?	__________ Rand OR<br>[ ] nothing OR<br>[ ] don't know	
**Traditional medicine**			
5.0	**The question concerns Period 1.** In period 1, how much money have you spent on traditional medicines?	__________ Rand OR<br>[ ] nothing OR<br>[ ] don't know	If the fee for medicine was given combined with a fixed fee, enter full value in 6.3
5.1	In period 1, how many times have you been to a traditional healer?	__________times OR<br>[ ] no times OR<br>[ ] don't know	**If "no times", skip to next section**
5.2	How long, on average, did you spend with this healer for one consultation in period 1, including the time needed for waiting on the day for your appointment?	____:____ hours OR<br>[ ] don't know	
5.3	How much have you paid in total across all your visits to a healer in period 1 in fixed charges or fees?	__________ Rand OR<br>[ ] nothing OR<br>[ ] don't know	
5.4	What is the method of transport you use most often to get to this healer in period 1?	car [ ]<br>bicycle [ ]<br>motorbike/scooter [ ]<br>by foot [ ]<br>by public transport [ ]<br>privately hired taxi [ ]	If public transport or hired taxi, proceed to 6.5. Other answers - skip to 6.6
5.5	If you took public transport or hired a taxi, how much did you spend, on average, for a journey to and from this healer?	__________ Rand OR<br>[ ] nothing OR<br>[ ] don't know	

5.6	How long does the journey take to go to and from your healer from home?	____:____ hours OR<br>[ ] don't know	
5.7	On average, how much did you need to spend on food on a single one of these visits to a healer in period 1?	__________ Rand OR<br>[ ] nothing OR<br>[ ] don't know	
5.8	How much, per visit, have you spent on accommodation that you needed because of all your visits to the healer in period 1?	__________ Rand OR<br>[ ] nothing OR<br>[ ] don't know	
5.9	On visits when you were accompanied, how many adults, on average, accompanied you to these visits? And how many of the visits in this period were they there for?	____ adults for _____visits<br>[ ] no one accompanied me OR<br>[ ] don't know	If no one accompnaied patient, please insert "0" and skip to next section
5.10	How long would another adult spend accompanying you to on an average visit to the traditional healer in period 1? Include **waiting time, appointment time and time taken for the return journey**	____:____ hours OR<br>[ ] no one accompanied me OR<br>[ ] don't know	
5.11	How much, per visit, has been spent on accommodation, food and transport that the main person accompanying you needed on these visits to a healer in period 1?	__________ Rand OR<br>[ ] nothing OR<br>[ ] don't know	
**Specialist doctors (usually in hospital outpatients; also private clinics, usually referred by another doctor)**			
6.0	This whole question concerns Period 1. In Period 1, how many times did you visit a specialist doctor?	__________times OR<br>[ ] no times OR<br>[ ] don't know	If "no times", skip to 8.0
6.1	How long, on average, did you spend **waiting for, and in,** an appointment you had at these clinics in period 1?	____:____ hours OR<br>[ ] don't know	
6.2	How much have you paid **for Xray/radiology tests** in total across all these visits in period 1 (excluding transport)?	__________ Rand OR<br>[ ] nothing OR<br>[ ] don't know	
6.3	How much have you paid for tests in total across all your visits that were **not** X rays/radiology in period 1, that were requested by speciality doctors?	__________ Rand OR<br>[ ] nothing OR<br>[ ] don't know	
6.4	How much have you paid in total across all your visits to speciality doctors' clinics in period 1 in fixed charges or fees?	__________ Rand OR<br>[ ] nothing OR<br>[ ] don't know	

6.5	How much did you spend on buying drugs from speciality doctors in total across all your visits to clinic in period 1?	__________ Rand OR<br>[ ] nothing OR<br>[ ] don't know	
6.6	What is the method of transport you use most often to get to these speciality doctors' appointments in period 1?	car [ ]<br>bicycle [ ]<br>motorbike/scooter [ ]<br>by foot [ ]<br>by public transport [ ]<br>privately hired taxi [ ]	If public transport or hired taxi, proceed to 7.7. Other answers - skip to 7.8
6.7	If you took public transport or hired a taxi, how much did you spend, on average, for a journey to and from this/these clinic(s)	__________ Rand OR<br>[ ] nothing OR<br>[ ] don't know	
6.8	How long does the journey take to go to and from the speciality clinic from home?	____:____ hours OR<br>[ ] don't know	
6.9	On average, how much did you need to spend on food on a single one of these visits in period 1?	__________ Rand OR<br>[ ] nothing OR<br>[ ] don't know	
6.10	How much, per visit, have you spent on accommodation that you needed because of your visits to the clinic(s) in period 1?	__________ Rand OR<br>[ ] nothing OR<br>[ ] don't know	
6.11	On visits when you were accompanied, how many adults, on average, accompanied you to these visits? And how many of the visits in this period were they there for?	____ adults for _____visits<br>[ ] no one accompanied me OR<br>[ ] don't know	If "no one accompanied me" then skip to 8.0
6.12	How long would another adult spend accompanying you to on an average visit to a specialist in period 1? Include **waiting time, appointment time and time taken for the return journey**	____:____ hours OR<br>[ ] don't know	
6.13	How much, per visit, has been spent on **accommodation, food and transport** that the main adult accompanying you needed because of these visits to a specialist in Period 1?	__________ Rand OR<br>[ ] nothing OR<br>[ ] don't know	
**Radiology transport**			
7.0	did you have to travel for X rays in period 1?	[ ] Yes, ____ times<br>[ ] No<br>[ ] don't know	If no,skip to 9.0
7.1	If yes then what is the method of transport you use most often to get to get X rays done in period 1?	car [ ]<br>bicycle [ ]<br>motorbike/scooter [ ]<br>by foot [ ]<br>by public transport [ ]<br>privately hired taxi [ ]	If "public transport", or if "privately hired car" continue to 8.2. If not, continue to 8.3

7.2	If you took public transport or hired a taxi, how much did you spend, on average, for a journey to and from the place where the Xrays were done	__________ Rand OR<br>[ ] nothing OR<br>[ ] don't know	
7.3	How long, on average, would you spend waiting for the x ray and getting it done?	__:___ hours<br>or<br>[ ] don't know	
7.4	How long does the journey take to go to and from the X ray department from home or clinic, depending on which journey you made?	____:____ hours OR<br>[ ] don't know	
7.5	On average, how much did you need to spend on food on a single one of these visits in period 1?	__________ Rand OR<br>[ ] nothing OR<br>[ ] don't know	
7.6	How much, per visit, have you spent on accommodation that you needed for these visits?	__________ Rand OR<br>[ ] nothing OR<br>[ ] don't know	
7.7	On visits when you were accompanied, how many adults, on average, accompanied you to these visits? And how many of the visits in this period were they there for?	____ adults for _____visits<br>[ ] no one accompanied me OR<br>[ ] don't know	If no one accompnaied patient, please insert "0" and skip to next section
7.8	How long would another adult spend accompanying you to on an average visit to get an x ray in period 1? Include **waiting time, appointment time and time taken for the return journey**	____:____ hours OR<br>[ ] don't know	
7.9	How much, per visit, has been spent on accommodation, food and transport that the main person accompanying you needed because of these visits to an X ray department in period 1?	__________ Rand OR<br>[ ] nothing OR<br>[ ] don't know	
**HEALTHCARE EXPENDITURE PERIOD 2**			
We are now going to talk about later periods on your timescale			
1.0	Have you been investigated for TB in any of the time periods on the timescale we have drawn?	[ ] Yes<br>[ ] No<br>[ ] Don't know	If no or don't know, proceed to 3.0. If yes, proceed to 2.0.
2.0	If yes, How long, roughly, was the time taken between your joining the study and the decision to be made by your doctors that you could **start your ART?**	__________ days OR<br>Don't know [ ]	date of **ART starting** minus date of enrolment
2.1	If yes to 1.0, were you diagnosed as being positive or negative for TB?	[ ] Positive<br>[ ] Negative<br>[ ] Don't know	

2.2	Did you receive any ART medications for HIV while you were still awaiting the results of your TB testing?	[ ] Yes<br>[ ] No<br>[ ] Don't know	
3.0	Did you request that starting either HIV or TB medications should be delayed, for any reason?	[ ] Yes<br>[ ] No<br>[ ] Don't know	If yes proceed to 3.1, **if no or don't know, skip to 4.0**
3.1	Which did you ask to delay?	[ ] ARVs<br>[ ] TB treatment<br>[ ] both	
3.2	How long was this delay?	__________ days OR<br>Don't know [ ]	
I am now going to ask you several questions about period 2. Even if the period was only a day long we would like to ask you about the trial clinic			
**Trial Clinic**			
4.0	This whole question concerns **Period 2.** In Period 2, how many times did you visit the trial clinic, including its emergency room?	_____ time for **Enrolment**+-compliance<br>_____ times for **Initiation** of treatment +-compliance OR<br>[ ] don't know<br>_____ times for **combined enrolment and initiation (in one day)**+-compliance OR<br>[ ] don't know<br>_____times for **lonestanding compliance visits** OR<br>[ ] don't know<br>_____times for HIV/TB checkups or investigations OR<br>[ ] don't know<br>_____times for other issues OR<br>[ ] don't know	Everyone should have been to this clinic once or twice in period 2 (enrolment and the visit to start TB Rx/ART if TB negative).<br><br>Lonestanding compliance visit = an appointment when you only had compliance counselling

4.1	How long, on average, did you spend waiting for, and in, an appointment you had at the trial clinic in Period 2? Also please include time spent eg. Waiting for medications, waiting for tests, booking further appointments	Enrolment visit+-compliance:<br>____:____ hours OR<br>[ ] don't know<br>Treatment initiation visit+-compliance:<br>____:____ hours OR<br>[ ] don't know<br>Combined enrolment and initiation+-compliance **(in one day):**<br>____:____ hours OR<br>[ ] don't know<br>Lone compliance visits: ____:____ hours<br>[ ] don't know<br>HIV/TB checkups or investigations:<br>____:____ hours OR<br>[ ] don't know<br>Other issues:<br>____:____ hours<br>[ ] don't know	please fill in "0:00" if patient did not have specified visit type
4.2	What is the method of transport you use most often to get to trial clinic appointments in Period 2?	car [ ]<br>bicycle [ ]<br>motorbike/scooter [ ]<br>by foot [ ]<br>by public transport [ ]<br>privately hired taxi [ ]	If public transport or hired taxi, proceed to 4.7. Other answers - skip to 4.8
4.3	If you took public transport or hired a taxi, and the journey had not been reimbursed to you, how much would you have spent, on average, for a journey to and from the trial clinic?	__________ Rand OR<br>[ ] nothing OR<br>[ ] don't know	
4.4	How long does the journey take to go to and from the trial clinic from home?	____:____ hours OR<br>[ ] don't know	
4.5	On average, how much did you need to spend on food on a single one of these trial clinic visits in Period 2?	__________ Rand OR<br>[ ] nothing OR<br>[ ] don't know	
4.6	On visits when you were accompanied, how many adults, on average, accompanied you to these visits? And how many of the visits in this period were they there for?	____ adults for _____visits<br>[ ] no one accompanied me OR<br>[ ] don't know	If "no one accompanied me" then skip to 5.0
4.7	How long would another adult spend accompanying you to on an average visit to the trial clinic in period 2? Include waiting time, appointment time and time taken for the return journey	____:____ hours OR<br>[ ] no one accompanied me OR<br>[ ] don't know	

4.8	How much, per visit, has been spent on accommodation, food and transport that the main person accompanying you needed because of these visits to the trial clinic in Period 2?	__________ Rand OR<br>[ ] nothing OR<br>[ ] don't know	IF patient's P2 was 1 day long, skip to "Health Expenditure Period 3" (unless had an X ray in P2 in which case please skip to "radiology transport")
**Nonspecialist clinic visits excluding trial clinic**			
5.0	This whole question concerns Period 2. In Period 2, how many times did you visit a medical clinic, excluding the trial clinic (not including a GP (private doctor)), and includingthe use of the emergency room in clinics?	__________times OR<br>[ ] no times OR<br>[ ] don't know	**If "no times", skip to 6.0**
5.1	How long, on average, did you spend **waiting for, and in**, an appointment you had at these clinics in Period 2? Also please include time spent eg. Waiting for medications, waiting for tests, booking further appointments	____:____ hours OR<br>[ ] don't know	
5.2	What is the method of transport you use most often to get to these clinic appointments in Period 2?	car [ ]<br>bicycle [ ]<br>motorbike/scooter [ ]<br>by foot [ ]<br>by public transport [ ]<br>privately hired taxi [ ]	If public transport or hired taxi, skip to 5.7. Other answers - proceed to 5.8
5.3	If you took public transport or hired a taxi, how much did you spend, on average, for a journey to and from this/these clinic(s)	__________ Rand OR<br>[ ] nothing OR<br>[ ] don't know	
5.4	How long does the journey take to go to and from your clinic from home?	____:____ hours OR<br>[ ] don't know	
5.5	On average, how much did you need to spend on food on a single one of these clinic visits in Period 2?	__________ Rand OR<br>[ ] nothing OR<br>[ ] don't know	
5.6	How much did you spend, per visit, on accommodation that you needed because of your visits to the clinic(s) in Period 2?	__________ Rand OR<br>[ ] nothing OR<br>[ ] don't know	
5.7	How much, in total over Period 2, do you think you spent on "under the table payments" at clinics?	__________ Rand OR<br>[ ] nothing OR<br>[ ] don't know	

5.8	On visits when you were accompanied, how many adults, on average, accompanied you to these visits? And how many of the visits in this period were they there for?	____ adults for _____visits<br>[ ] no one accompanied me OR<br>[ ] don't know	If "no one accompanied me" then skip to 6.0
5.9	How long would another adult spend accompanying you to on an average visit to these clinics in period 2? Include **waiting time, appointment time and time taken for the return journey**	____:____ hours OR<br>[ ] no one accompanied me OR<br>[ ] don't know	
5.10	How much, per visit, has been spent on accommodation, food and transport that the main person accompanying you needed because of these visits to clinic in period 2?	__________ Rand OR<br>[ ] nothing OR<br>[ ] don't know	
	**Hospital/Hospice inpatient**		
6.0	This whole question concerns Period 2. In Period 2, how many times, if any, were you admitted to hospital or to a hospice?	__________times OR<br>[ ] no times OR<br>[ ] don't know	**If "no times", skip to 7.0**
6.1	How long was/were your admissions to hospital or a hospice, in Period 2?	Visit 1: _________ days<br>Visit 2: _________days<br>Visit 3:_________days<br>[ ] don't know	
6.2	How much did you pay in fixed charges or fees on your last hospital or hospice admission in Period 2, including "opening a file"?	__________ Rand OR<br>[ ] nothing OR<br>[ ] can't remember	
6.3	What is the method of transport you used to get to your last hospital or hospice admission in Period 2?	car [ ]<br>bicycle [ ]<br>motorbike/scooter [ ]<br>by foot [ ]<br>by public transport [ ]<br>privately hired taxi [ ]<br>ambulance [ ]	If **public transport**, hired taxi, or **ambulance** proceed to 6.8. other answers - skip to 6.9
6.4	If you took public transport or hired a taxi or an ambulance, how much did you spend for a journey to and from the hospital or hospice?	__________ Rand OR<br>[ ] nothing OR<br>[ ] don't know	
6.5	How long does the journey take to go to and from hospital or the hospice from home?	____:____ hours OR<br>[ ] don't know	
6.6	On your hospital or hospice admission in period 2, approximately how many days did you have one or more visitors present?	_________ days OR<br>[ ] no-one accompanied me<br>[ ] don't know	If "no one accompanied me" then skip to 7.0

6.7	On days when you had visitors, how many visitors did you have, on average?	__________ visitors OR<br>[ ] don't know	
6.8	On average, on days when you had a visitor, how many **hours** did your visitor spend **visiting you and on the journey time for the return journey** to and from hospital or the hospice on a **single visit** in period 2?	____:____ hours OR<br>[ ] don't know	
6.9	How much, if anything, was spent on accommodation that that person needed because of your last hospital or hospice admission in period 2?	___________ Rand OR<br>[ ] nothing OR<br>[ ] don't know	
6.10	How much, per visit, was spent on food and transport that your visitor needed to eat while accompanying you during this admission to hospital or hospice in period 2?	___________ Rand OR<br>[ ] nothing OR<br>[ ] don't know	
	**GPs (Private Doctors)**		
7.0	This question concerns Period 2. In Period 2, how many visits did you make to your GP (Private Doctor)?	___________times OR<br>[ ] no times OR<br>[ ] don't know	**If "no times", skip to 9.0**
7.1	How long, on average, did you spend waiting for, and in, an appointment you had at your GP (private doctor) in Period 2? Also please include time spent eg. Waiting for tests, booking further appointments	____:____ hours OR<br>[ ] don't know	
7.2	How much have you paid in total for all Xray/radiology tests that your GP (private doctor) requested for you in total across all your visits to him/her in Period 2?	___________ Rand OR<br>[ ] nothing OR<br>[ ] don't know	
7.3	How much have you paid for tests from your GP (private doctor) in total across all your visits that were not X rays/radiology in Period 2?	___________ Rand OR<br>[ ] nothing OR<br>[ ] don't know	
7.4	How much have you paid in fixed charges or fees in total in visits to yor family doctor in Period 2?	___________ Rand OR<br>[ ] nothing OR<br>[ ] don't know	

7.5	What is the method of transport you use most often to get to these GP (private doctor) appointments in Period 2?	car [ ]<br>bicycle [ ]<br>motorbike/scooter [ ]<br>by foot [ ]<br>by public transport [ ]<br>privately hired taxi [ ]	If public transport or hired taxi, proceed to 8.6. Other answers - skip to 8.7
7.6	If you took public transport or hired a taxi, how much did you spend, on average, for a journey to and from your GP (private doctor) in Period 2?	__________ Rand OR<br>[ ] nothing OR<br>[ ] don't know	
7.7	How long does the journey take to go to and from your GP (private doctor) from home?	____:____ hours OR<br>[ ] don't know	
7.8	On average, how much did you need to spend on food on a single one of these GP (private doctor) visits in Period 2?	__________ Rand OR<br>[ ] nothing OR<br>[ ] don't know	
7.9	On visits when you were accompanied, how many adults, on average, accompanied you to these visits? And how many of the visits in this period were they there for?	____ adults for _____visits<br>[ ] no one accompanied me OR<br>[ ] don't know	If "no one accompanied me" then skip to 9.0
7.10	How long would another adult spend accompanying you to on an average visit to a GP (private doctor) in period 1? Include waiting time, appointment time and time taken for the return journey	____:____ hours OR<br>[ ] no one accompanied me OR<br>[ ] don't know	
7.11	How much, per visit, has been spent on accommodation, food and transport that the main adult accompanying you needed because of these visits to the GP (private doctor) in Period 2?	__________ Rand OR<br>[ ] nothing OR<br>[ ] don't know	
	**Pharmacies (not in a hospital or clinic, not a traditional healer)**		
8.0	This question is about Period 2. In Period 2, how many times did you go to a pharmacy because of your health?	__________times OR<br>[ ] no times OR<br>[ ] don't know	
8.1	In Period 2, how many times did someone else go to a pharmacy because of your health?	__________times OR<br>[ ] no times OR<br>[ ] don't know	**If "no times"** is answer to to 9.0 **and** 9.1**, skip to 10.0, otherwise proceed to 9.2**
8.2	What is the method of transport you (or the person going to pharmacy for you) used most often to get to a pharmacy in Period 2?	car [ ]<br>bicycle [ ]<br>motorbike/scooter [ ]<br>by foot [ ]<br>by public transport [ ]<br>privately hired taxi [ ]	If public transport or hired taxi, proceed to 9.3. Other answers - skip to 9.4

8.3	If you (or the person going to pharmacy for you) took public transport or hired a taxi, how much did you spend, on average, for a journey to and from this pharmacy?	__________ Rand OR<br>[ ] nothing OR<br>[ ] don't know	
8.4	What was the total cost of all the drugs you bought in pharmacies in period 2, do you think?	__________ Rand OR<br>[ ] nothing OR<br>[ ] don't know	
8.5	How long does the journey take to go to and from your pharmacy from home, including waiting time for the drugs to be made ready?	____:____ hours OR<br>[ ] don't know	
8.6	On average, how much did you or the person going for you need to spend on food on a single one of these pharmacy visits in Period 2?	__________ Rand OR<br>[ ] nothing OR<br>[ ] don't know	
	**Traditional medicine**		
9.0	**The question concerns Period 2.** In period 2, how much money have you spent on traditional medicines?	__________ Rand OR<br>[ ] nothing OR<br>[ ] don't know	If the fee for medicine was given **combined** with a fixed fee, enter full value in 10.3
9.1	In Period 2, how many times have you been to a traditional healer?	__________times OR<br>[ ] no times OR<br>[ ] don't know	**If "no times", skip to 11**
9.2	How long, on average, did you spend with this healer for one visit in period 2, including the time needed for waiting on the day for your appointment?	____:____ hours OR<br>[ ] don't know	
9.3	How much have you paid in total across all your visits to a healer in Period 2 in fixed charges or fees?	__________ Rand OR<br>[ ] nothing OR<br>[ ] don't know	
9.4	What is the method of transport you use most often to get to this healer in Period 2?	car [ ]<br>bicycle [ ]<br>motorbike/scooter [ ]<br>by foot [ ]<br>by public transport [ ]<br>privately hired taxi [ ]	If public transport or hired taxi, proceed to 10.5. Other answers - skip to 10.6
9.5	If you took public transport or hired a taxi, how much did you spend, on average, for a journey to and from this healer?	__________ Rand OR<br>[ ] nothing OR<br>[ ] don't know	
9.6	How long does the journey take to go to and from your healer from home?	____:____ hours OR<br>[ ] don't know	
9.7	On average, how much did you need to spend on food on a single one of these visits to a healer in Period 2?	__________ Rand OR<br>[ ] nothing OR<br>[ ] don't know	

9.8	How much per visit have you spent on accommodation that you needed when visiting the healer in Period 2?	__________ Rand OR<br>[ ] nothing OR<br>[ ] don't know	
9.9	On visits when you were accompanied, how many adults, on average, accompanied you to these visits? And how many of the visits in this period were they there for?	____ adults for _____visits<br>[ ] no one accompanied me OR<br>[ ] don't know	If "no one accompanied me" then skip to 11.0
9.10	How long would another adult spend accompanying you to on an average visit to the healer in period 2? Include **waiting time, appointment time and time taken for the return journey**	____:____ hours OR<br>[ ] no one accompanied me OR<br>[ ] don't know	
9.11	How much, per visit, has been spent on **accommodation, food and transport** that the main adult accompanying you needed because of these visits to the healer in Period 2?	__________ Rand OR<br>[ ] nothing OR<br>[ ] don't know	
**Specialist doctors (usually in hospital outpatients; also private clinics, usually referred by another doctor)**			
10.0	This whole question concerns Period 2. In Period 2, how many times did you visit a specialist doctor?	__________times OR<br>[ ] no times OR<br>[ ] don't know	If "no times", proceed to 12.0
10.1	How long, on average, did you spend **waiting for, and in,** an appointment you had at these clinics in Period 2?	____:____ hours OR<br>[ ] don't know	
10.2	How much have you paid for Xray/radiology tests in total across all these visits in Period 2? (excluding transport)	__________ Rand OR<br>[ ] nothing OR<br>[ ] don't know	
10.3	How much have you paid for tests in total across all your visits that were not X rays/radiology in Period 2, that were requested by speciality doctors?	__________ Rand OR<br>[ ] nothing OR<br>[ ] don't know	
10.4	How much have you paid in total across all your visits to speciality doctors' clinics in Period 2 in fixed charges or fees?	__________ Rand OR<br>[ ] nothing OR<br>[ ] don't know	
10.5	What is the method of transport you use most often to get to these speciality doctors' appointments in Period 2?	car [ ]<br>bicycle [ ]<br>motorbike/scooter [ ]<br>by foot [ ]<br>by public transport [ ]<br>privately hired taxi [ ]	If public transport or hired taxi, proceed to 11.7. Other answers - skip to 6.8

10.6	If you took public transport or hired a taxi, how much did you spend, on average, for a journey to and from this/these clinic(s)	__________ Rand OR<br>[ ] nothing OR<br>[ ] don't know	
10.7	How long does the journey take to go to and from the speciality clinic from home?	____:____ hours OR<br>[ ] don't know	
10.8	On average, how much did you need to spend on food on a single one of these visits in Period 2?	__________ Rand OR<br>[ ] nothing OR<br>[ ] don't know	
10.9	How much, per visit, have you spent on accommodation that you needed because of your visits to the clinic(s) in Period 2?	__________ Rand OR<br>[ ] nothing OR<br>[ ] don't know	
10.10	On visits when you were accompanied, how many adults, on average, accompanied you to these visits? And how many of the visits in this period were they there for?	____ adults for _____visits<br>[ ] no one accompanied me OR<br>[ ] don't know	If "no one accompanied me" then skip to 12.0
10.11	How long would another adult spend accompanying you to on an average visit to these clinics in period 2? Include **waiting time, appointment time and time taken for the return journey**	____:____ hours OR<br>[ ] no one accompanied me OR<br>[ ] don't know	
10.12	How much, per visit, has been spent on **accommodation, food and transport** that the main adult accompanying you needed because of these visits to these clinics in Period 2?	__________ Rand OR<br>[ ] nothing OR<br>[ ] don't know	
	**Radiology transport**		
11.0	did you have to travel for X rays in Period 2?	[ ] Yes, ____ times<br>[ ] No<br>[ ] don't know	If no,skip to "Healthcare expenditure period 3"
11.1	If yes then what is the method of transport you use most often to get to get X rays done in Period 2?	car [ ]<br>bicycle [ ]<br>motorbike/scooter [ ]<br>by foot [ ]<br>by public transport [ ]<br>privately hired taxi [ ]	If public transport, continue to 12.2, if no public transport skip to 12.3
11.2	If you took public transport or hired a taxi, how much did you spend, on average, for a journey to and from the place where the Xrays were done	__________ Rand OR<br>[ ] nothing OR<br>[ ] don't know	
11.3	How long, on average, would you spend waiting for the x ray and getting it done?	__:___ hours<br>or<br>[ ] don't know	

11.4	How long does the journey take to go to and from the X ray department from home or clinic, depending on which journey you made?	____:____ hours OR<br>[ ] don't know	
11.5	On average, how much did you need to spend on food on a single one of these visits in Period 2?	__________ Rand OR<br>[ ] nothing OR<br>[ ] don't know	
11.6	On visits when you were accompanied, how many adults, on average, accompanied you to these visits? And how many of the visits in this period were they there for?	____ adults for _____visits<br>[ ] no one accompanied me OR<br>[ ] don't know	If "no one accompanied me" then skip to 13.0
11.7	How long would another adult spend accompanying you to on an average visit to ____ in period ____? Include **waiting time, appointment time and time taken for the return journey**	____:____ hours OR<br>[ ] no one accompanied me OR<br>[ ] don't know	
11.8	How much, per visit, has been spent on accommodation, food and transport that the main person accompanying you needed because of these visits to an X ray department in Period 2?	__________ Rand OR<br>[ ] nothing OR<br>[ ] don't know	
**HEALTHCARE EXPENDITURE PERIOD 3**			
	I am now going to ask you several questions about Period 3		
**Trial Clinic**			
1.0	This whole question concerns Period 3. In Period 3, on how many occasions did you visit the trial clinic, including to the emergency room in the trial clinic? **Do not include occasions when you were visiting the trial nurse only**	**HIV treatment/tests/medicines (including days in which had TB/HIV on same day)**:<br>_____times OR [ ] don't know<br>**TB treatment/tests/medicines (including days in which had TB/HIV on same day):**<br>_____times OR [ ] don't know<br>**Did** you have any days where you had a TB and an HIV appointment on the **same day? If so, how many? ______**<br>**Compliance visits:**<br>_____times OR [ ] don't know<br>**Other clinic appointments**<br>_____times OR [ ] don't know	please say "00.00" if no visits<br><br>NB if a patient **cannot recall how long** the ART vs the HIV clinics were in length, but can remember on

1.1	How long, on average, did you spend **waiting for, and in,** an appointment you had at the trial clinic in Period 3? Also please include time spent eg. Waiting for medications, waiting for tests, booking further appointments. Please **exclude any hours spent with or waiting for trial nurses**	**HIV treatment/tests/medicines (including days in which had TB/HIV on same day)**:<br>___:___ hours OR [ ] don't know<br>**TB treatment/tests/medicines (including days in which had TB/HIV on same day):**<br>___:___ hours OR [ ] don't know<br>**How long** were clinic days where you had a TB and an HIV appointment on the **same day? ___:___ hours**<br>**Compliance visits:**<br>___:___ hours OR [ ] don't know<br>**Other clinic appointments**<br>___:___ hours OR [ ] don't know	[illegible] average how long they were in clinic **please insert this into the "other"** space. Also **use this space where pt attended 2 clinics on the same day**
1.2	What is the method of transport you use most often to get to trial clinic appointments in Period 3?	car [ ]<br>bicycle [ ]<br>motorbike/scooter [ ]<br>by foot [ ]<br>by public transport [ ]<br>privately hired taxi [ ]	If public transport or hired taxi, proceed to 1.7. Other answers - skip to 1.8
1.3	If you took public transport or hired a taxi, and the journey had not been reimbursed to you, how much would you have spent, on average, for a journey to and from the trial clinic?	__________ Rand OR<br>[ ] nothing OR<br>[ ] don't know	
1.4	How long does the journey take to go to and from the trial clinic from home?	____:____ hours OR<br>[ ] don't know	
1.5	On average, how much did you need to spend on food on a single one of these trial clinic visits in Period 3?	__________ Rand OR<br>[ ] nothing OR<br>[ ] don't know	
1.6	How much did you spend, per visit, on accommodation that you needed because of your visits to the trial clinic in Period 3?	__________ Rand OR<br>[ ] nothing OR<br>[ ] don't know	
1.7	On visits when you were accompanied, how many adults, on average, accompanied you to these visits? And how many of the visits in this period were they there for?	____ adults for _____visits<br>[ ] no one accompanied me OR<br>[ ] don't know	If "no one accompanied me" then skip to 2.0
1.8	How long would another adult spend accompanying you to on an average visit to the trial clinic in period 3? Include **waiting time, appointment time and time taken for the return journey**	____:____ hours OR<br>[ ] no one accompanied me OR<br>[ ] don't know	

1.9	How much, per visit, has been spent on accommodation, food and transport that the main person accompanying you needed because of these visits to the trial clinic in Period 3?	__________ Rand OR<br>[ ] nothing OR<br>[ ] don't know	
	**Nonspecialist clinic visits excluding trial clinic**		
2.0	This whole question concerns Period 3. In Period 3, how many times did you visit a medical clinic, not including a GP (private doctor), directly observed therapy (DOTS) facility or trial clinic (including emergency room visits in clinics)?	__________times OR<br>[ ] no times OR<br>[ ] don't know	If "no times", skip to 3.0. This defnition of outpatient clinic applies through all of question 1
2.1	How long, on average, did you spend **waiting for, and in**, an appointment you had at these clinics in Period 3? Also please include time spent eg. Waiting for medications, waiting for tests, booking further appointments	____:____ hours OR<br>[ ] don't know	
2.2	What is the method of transport you use most often to get to these clinic appointments in Period 3?	car [ ]<br>bicycle [ ]<br>motorbike/scooter [ ]<br>by foot [ ]<br>by public transport [ ]<br>privately hired taxi [ ]	If public transport or hired taxi, proceed to 2.7. Other answers - skip to 2.8
2.3	If you took public transport or hired a taxi, how much did you spend, on average, for a journey to and from this/these clinic(s)	__________ Rand OR<br>[ ] nothing OR<br>[ ] don't know	
2.4	How long does the journey take to go to and from your clinic from home?	____:____ hours OR<br>[ ] don't know	
2.5	On average, how much did you need to spend on food on a single one of these clinic visits in Period 3?	__________ Rand OR<br>[ ] nothing OR<br>[ ] don't know	
2.6	On visits when you were accompanied, how many adults, on average, accompanied you to these visits? And how many of the visits in this period were they there for?	____ adults for _____visits<br>[ ] no one accompanied me OR<br>[ ] don't know	If "no one accompanied me" then skip to 3.0

2.7	How long would another adult spend accompanying you to on an average visit to the trial clinic in period 3? Include **waiting time, appointment time and time taken for the return journey**	____:____ hours OR<br>[ ] no one accompanied me OR<br>[ ] don't know	
2.8	How much, per visit, has been spent on **accommodation, food and transport** that the main adult accompanying you needed because of these visits to the trial clinic in Period 3?	__________ Rand OR<br>[ ] nothing OR<br>[ ] don't know	
	**Hospital/hospice admissions (inpatient)**		
3.0	This whole question concerns Period 3. In Period 3, how many times, if any, were you admitted to **hospital or a hospice**?	__________times OR<br>[ ] no times OR<br>[ ] don't know	**If "no times", skip to 4.0**
3.1	How long was/were your admissions to hospital or a hospice, in Period 3?	Visit 1: _________ days<br>Visit 2: _________days<br>Visit 3:_________days<br>[ ] don't know	
3.2	How much did you pay in fixed charges or fees on your last hospital or hospice admission in Period 3 including "opening a file"?	__________ Rand OR<br>[ ] nothing OR<br>[ ] can't remember	
3.3	How much did you spend paying for a bed and "hotel services" including food on your last hospital or hospice admission in Period 3?	__________ Rand OR<br>[ ] nothing OR<br>[ ] don't know	
3.4	What is the method of transport you used to get to your last hospital or hospice admission in Period 3?	car [ ]<br>bicycle [ ]<br>motorbike/scooter [ ]<br>by foot [ ]<br>by public transport [ ]<br>privately hired taxi [ ]<br>ambulance [ ]	If **public transport**, **privately hired taxi** or **ambulance** proceed to 3.8. other answers - skip to 3.9
3.5	If you took public transport or hired a taxi or an ambulance, how much did you spend for a journey to and from hospital or the hospice?	__________ Rand OR<br>[ ] nothing OR<br>[ ] don't know	
3.6	How long does the journey take to go to and from hospital or the hospice from home?	____:____ hours OR<br>[ ] don't know	
3.7	On your hospital or hospice admission in period 3, approximately how many days did you have one or more visitors present?	_________ days OR<br>[ ] no-one accompanied me<br>[ ] don't know	If "no one accompanied me" then skip to 4.0

3.8	On days when you had visitors, how many visitors did you have, on average?	__________ visitors OR<br>[ ] don't know	
3.9	On average, on days when you had a visitor, how many **hours** did your visitor spend **visiting you and on the journey time for the return journey** to and from hospital or the hospice on a **single visit** in period 3?	____:____ hours OR<br>[ ] don't know	
3.10	How much, if anything, was spent on accommodation that that person needed because of your last hospital or hospice admission in period 3?	__________ Rand OR<br>[ ] nothing OR<br>[ ] don't know	
3.11	How much, per visit, was spent on food and transport that your visitor needed to eat while accompanying you during this admission to hospital or to a hospice in period 3?	__________ Rand OR<br>[ ] nothing OR<br>[ ] don't know	
	**GPs (Private Doctor)**		
4.0	This question concerns Period 3. In Period 3, how many visits did you make to your private doctor (General Practitioner)?	__________times OR<br>[ ] no times OR<br>[ ] don't know	**If "no times", skip to 6.0**
4.1	How long, on average, did you spend waiting for, and in, an appointment you had at your GP (private doctor) in Period 3? Also please include time spent eg. Waiting for medications, waiting for tests, booking further appointments	____:____ hours OR<br>[ ] don't know	
4.2	How much have you paid in total for all Xray/radiology tests that your GP (private doctor) requested for you in total across all your visits to him/her in Period 3?	__________ Rand OR<br>[ ] nothing OR<br>[ ] don't know	
4.3	How much have you paid for tests from your GP (private doctor) in total across all your visits that were not X rays/radiology in Period 3?	__________ Rand OR<br>[ ] nothing OR<br>[ ] don't know	
4.4	How much have you paid in fixed charges or fees in total in visits to yor fmaily doctor in Period 3?	__________ Rand OR<br>[ ] nothing OR<br>[ ] don't know	

4.5	What is the method of transport you use most often to get to these GP (private doctor) appointments in Period 3?	car [ ]<br>bicycle [ ]<br>motorbike/scooter [ ]<br>by foot [ ]<br>by public transport [ ]<br>privately hired taxi [ ]	If public transport or hired taxi, proceed to 5.6. Other answers - skip to 5.7
4.6	If you took public transport or hired a taxi, how much did you spend, on average, for a journey to and from your GP (private doctor) in Period 3?	__________ Rand OR<br>[ ] nothing OR<br>[ ] don't know	
4.7	How long does the journey take to go to and from your GP (private doctor) from home?	____:____ hours OR<br>[ ] don't know	
4.8	On average, how much did you need to spend on food on a single one of these GP (private doctor) visits in Period 3?	__________ Rand OR<br>[ ] nothing OR<br>[ ] don't know	
4.9	On visits when you were accompanied, how many adults, on average, accompanied you to these visits? And how many of the visits in this period were they there for?	____ adults for _____visits<br>[ ] no one accompanied me OR<br>[ ] don't know	If "no one accompanied me" then skip to 6.0
4.10	How long would another adult spend accompanying you to on an average visit to the GP (private doctor) in period 3? Include waiting time, appointment time and time taken for the return journey	____:____ hours OR<br>[ ] no one accompanied me OR<br>[ ] don't know	
4.11	How much, per visit, has been spent on accommodation, food and transport that the main adult accompanying you needed because of these visits to the GP (private doctor) in Period 3?	__________ Rand OR<br>[ ] nothing OR<br>[ ] don't know	
	**Pharmacies (not in a hospital or clinic, not a traditional healer)**		
5.0	This question is about Period 3. In Period 3, how many times did you go to a pharmacy because of your health?	__________times OR<br>[ ] no times OR<br>[ ] don't know	**If "no times"** is answer to to 6.0 **and** 6.1**, skip to 7.0, otherwise proceed to 6.2**
5.1	In Period 3, how many times did someone else go to a pharmacy because of your health?	__________times OR<br>[ ] no times OR<br>[ ] don't know	
5.2	What is the method of transport you (or the person going to pharmacy for you) used most often to get to a pharmacy in Period 3?	car [ ]<br>bicycle [ ]<br>motorbike/scooter [ ]<br>by foot [ ]<br>by public transport [ ] privately hired taxi [ ]	If public transport or hired taxi, proceed to 6.3. Other answers - skip to 6.4

5.3	How much, in total, have you spent on drugs from a pharmacy in period 3?	__________ Rand OR<br>[ ] nothing OR<br>[ ] don't know	
5.4	If you (or the person going to pharmacy for you) took public transport or hired a taxi, how much did you spend, on average, for a journey to and from this pharmacy?	__________ Rand OR<br>[ ] nothing OR<br>[ ] don't know	
5.5	How long does the journey take to go to and from your pharmacy from home, including waiting time for the drugs to be made ready?	____:____ hours OR<br>[ ] don't know	
5.6	On average, how much did you or the person going for you need to spend on food on a single one of these pharmacy visits in Period 3?	__________ Rand OR<br>[ ] nothing OR<br>[ ] don't know	
**Traditional medicine**			
6.0	**This question concerns Period 3.** In period 3, how much money have you spent on traditional medicines?	__________ Rand OR<br>[ ] nothing OR<br>[ ] don't know	If the fee for medicine was given combined with a fixed fee, enter full value in 7.3
6.1	In Period 3, how many times have you been to a traditional healer?	__________times OR<br>[ ] no times OR<br>[ ] don't know	**If "no times", skip to 8.0**
6.2	How long, on average, did you spend with this healer for one visit in Period 3, including the time needed for waiting on the day for your appointment?	____:____ hours OR<br>[ ] don't know	
6.3	How much have you paid in total across all your visits to a healer in Period 3 in fixed charges or fees?	__________ Rand OR<br>[ ] nothing OR<br>[ ] don't know	
6.4	What is the method of transport you use most often to get to this healer in Period 3?	car [ ]<br>bicycle [ ]<br>motorbike/scooter [ ]<br>by foot [ ]<br>by public transport [ ] privately hired taxi [ ]	If public transport or hired taxi, proceed to 7.6. Other answers - skip to 7.6
6.5	If you took public transport or hired a taxi, how much did you spend, on average, for a journey to and from this healer?	__________ Rand OR<br>[ ] nothing OR<br>[ ] don't know	
6.6	How long does the journey take to go to and from your healer from home?	____:____ hours OR<br>[ ] don't know	
6.7	On average, how much did you need to spend on food on a single one of these visits to a healer in Period 3?	__________ Rand OR<br>[ ] nothing OR<br>[ ] don't know	

6.8	How much, per visit, have you spent on accommodation that you needed because of all your visits to the healer in Period 3?	__________ Rand OR<br>[ ] nothing OR<br>[ ] don't know	
6.9	On visits when you were accompanied, how many adults, on average, accompanied you to these visits? And how many of the visits in this period were they there for?	____ adults for _____visits<br>[ ] no one accompanied me OR<br>[ ] don't know	If "no one accompanied me" then skip to 8.0
6.10	How long would another adult spend accompanying you to on an average visit to the healer in period 3? Include **waiting time, appointment time and time taken for the return journey**	____:____ hours OR<br>[ ] no one accompanied me OR<br>[ ] don't know	
6.11	How much, per visit, has been spent on accommodation, food and transport that the main person accompanying you needed because of these visits to a healer in Period 3?	__________ Rand OR<br>[ ] nothing OR<br>[ ] don't know	
**DOTS**			
7.0	If you have been treated for TB in Period 3, did you have to go to a facility to be observed taking you medications for TB (Directly Observed Therapy)?	[ ] I have never received treatment for TB<br>[ ] Yes<br>[ ] No<br>[ ] Don't know	If I have never received treatment, No, or not sure, skip to 9.0
7.1	If yes, how many visits per week did you make for Directly Observed Therapy	__________times	
7.2	What is the method of transport you use most often to get to these DOTS appointments in Period 3?	car [ ]<br>bicycle [ ]<br>motorbike/scooter [ ]<br>by foot [ ]<br>by public transport [ ] privately hired taxi [ ]	If public transport or hired taxi, proceed to 8.3. Other answers - skip to 8.4
7.3	If you took public transport or hired a taxi, how much did you spend, on average, for a journey to and from this DOTS facility?	__________ Rand OR<br>[ ] nothing OR<br>[ ] don't know	
7.4	How long does the journey take to go to and from that DOTS facility from home?	____:____ hours OR<br>[ ] don't know	
7.5	How long, on average, did you spend waiting your turn, and in your DOTS appointment in Period 3?	____:____ hours OR<br>[ ] don't know	
7.6	On average, how much did you need to spend on food on a single one of these DOTS facility visits in Period 3?	__________ Rand OR<br>[ ] nothing OR<br>[ ] don't know	

7.7	On visits when you were accompanied, how many adults, on average, accompanied you to these visits? And how many of the visits in this period were they there for?	____ adults for _____visits<br>[ ] no one accompanied me OR<br>[ ] don't know	If "no one accompanied me" then skip to 9.0
7.8	How long would another adult spend accompanying you to on an average visit to DOTS in period 3? Include **waiting time, appointment time and time taken for the return journey**	____:____ hours OR<br>[ ] no one accompanied me OR<br>[ ] don't know	
7.9	How much, per visit, has been spent on **accommodation, food and transport** that the main adult accompanying you needed because of these visits to the DOTS facility in Period 3?	__________ Rand OR<br>[ ] nothing OR<br>[ ] don't know	
**Specialist doctors (usually in hospital outpatients; also private clinics, usually referred by another doctor)**			
8.0	This whole question concerns Period 3. In Period 3, how many times did you visit a specialist doctor?	__________times OR<br>[ ] no times OR<br>[ ] don't know	If "no times", skip to 10.0
8.1	How long, on average, did you spend **waiting for, and in,** an appointment you had at these clinics in Period 3?	____:____ hours OR<br>[ ] don't know	
8.2	How much have you paid for Xray/radiology tests in total across all these visits in Period 3 (excluding transport)?	__________ Rand OR<br>[ ] nothing OR<br>[ ] don't know	
8.3	How much have you paid for tests in total across all your visits that were not X rays/radiology in Period 3, that were reuested by speciality doctors?	__________ Rand OR<br>[ ] nothing OR<br>[ ] don't know	
8.4	How much have you paid in total across all your visits to speciality doctors' clinics in Period 3 in fixed charges or fees?	__________ Rand OR<br>[ ] nothing OR<br>[ ] don't know	
8.5	What is the method of transport you use most often to get to these speciality doctors' appointments in Period 3?	car [ ]<br>bicycle [ ]<br>motorbike/scooter [ ]<br>by foot [ ]<br>by public transport [ ] privately hired taxi [ ]	If public transport or hired taxi, proceed to 6.7. Other answers - skip to 6.8
8.6	If you took public transport or hired a taxi, how much did you spend, on average, for a journey to and from this/these clinic(s)	__________ Rand OR<br>[ ] nothing OR<br>[ ] don't know	

8.7	How long does the journey take to go to and from the speciality clinic from home?	____:____ hours OR<br>[ ] don't know	
8.8	On average, how much did you need to spend on food on a single one of these visits in Period 3?	__________ Rand OR<br>[ ] nothing OR<br>[ ] don't know	
8.9	On visits when you were accompanied, how many adults, on average, accompanied you to these visits? And how many of the visits in this period were they there for?	____ adults for _____visits<br>[ ] no one accompanied me OR<br>[ ] don't know	If "no one accompanied me" then skip to 10.0
8.10	On average, how long would another adult spend accompanying you on a visit, including their journey time, in Period 3?	____:____ hours OR<br>[ ] no one accompanied me OR<br>[ ] don't know	
8.11	How much, per visit, has been spent on accommodation, food and transport that the main person accompanying you needed because of these visits to specialty doctors in Period 3?	__________ Rand OR<br>[ ] nothing OR<br>[ ] don't know	
	**Radiology transport**		
9.0	did you have to travel for X rays in Period 3?	[ ] Yes, ____ times<br>[ ] No<br>[ ] don't know	If no,skip to next section
9.1	If yes then what is the method of transport you use most often to get to get X rays done in Period 3?	car [ ]<br>bicycle [ ]<br>motorbike/scooter [ ]<br>by foot [ ]<br>by public transport [ ]<br>privately hired taxi [ ]	If public transport, or privately hired taxi, proceed to 10.2. If not public transport or privately hired taxi skip to 10.3
9.2	If you took public transport or hired a taxi, how much did you spend, on average, for a journey to and from the place where the Xrays were done	__________ Rand OR<br>[ ] nothing OR<br>[ ] don't know	
9.3	How long, on average, would you spend waiting for the x ray and getting it done?	__:___ hours<br>or<br>[ ] don't know	
9.4	How long does the journey take to go to and from the X ray department from home or clinic, depending on which journey you made?	____:____ hours OR<br>[ ] don't know	
9.5	On average, how much did you need to spend on food on a single one of these visits in Period 3?	__________ Rand OR<br>[ ] nothing OR<br>[ ] don't know	

9.6	On visits when you were accompanied, how many adults, on average, accompanied you to these visits? And how many of the visits in this period were they there for?	____ adults for _____visits<br>[ ] no one accompanied me OR<br>[ ] don't know	If "no one accompanied me" then skip to 11.0
9.7	On average, how long would another adult spend accompanying you on a visit, including their journey time, in Period 3?	____:____ hours OR<br>[ ] no one accompanied me OR<br>[ ] don't know	
9.8	How much, per visit, has been spent on accommodation, food and transport that the main person accompanying you needed because of these visits to an X ray department in Period 3?	__________ Rand OR<br>[ ] nothing OR<br>[ ] don't know	
**INCOME**			
**NB - "start of a period" can be seen as the first half of a period, and the "end of a period" as its second half. For the start of period 1 it is best considered to be the time when the patient had relatively few symptoms (or even the period just before period 1 whenthey had no symptoms).**			
**Personal monetary income**			
1.0	How money did you usually earn in a month, at the start of Period 1, **excluding odd/piece-jobs**?	__________ Rand/month OR<br>[ ] nothing OR<br>[ ] don't know<br>[ ] n/a (eg if homemaker)	NB please multiply weekly salaries by **4.5** to obtain monethly ones
1.1	...and at the end of period 1?	__________ Rand/month OR<br>[ ] nothing OR<br>[ ] don't know<br>[ ] n/a (eg if homemaker)	
1.2	...and at the start of Period 3?	__________ Rand/month OR<br>[ ] nothing OR<br>[ ] don't know<br>[ ] n/a (eg if homemaker)	
1.3	…and at the end of Period 3?	__________ Rand/month OR<br>[ ] nothing OR<br>[ ] don't know<br>[ ] n/a (eg if homemaker)	
1.4	Have you gained or lost employment because of your illness?	P1 Y[ ] N[ ] DK [ ]<br>P2 Y[ ] N[ ] DK [ ]<br>P3 Y[ ] N[ ] DK [ ]	Yes Gained=1 Yes Lost=2. Skip to 2.0 if no changes in earnings **in any period**

1.5	If there were changes in your personal income level over period 1, that were **not caused by** loss/gain of a job, were they because of your illness - please tick the one that **most applies?**	[ ] yes, related to how ill/well I felt<br>[ ] yes, related to time spent seeking healthcare<br>[ ] no, unrelated to my illness<br>[ ] I don't know<br>[ ] my income only changed because I lost/gained a job<br>[ ] N/A	
1.6	If there were changes in your personal income level over period 2, that were **not caused by loss/gain of a job**, were they because of your illness - please tick the one that **most applies**?	[ ] yes, related to how ill/well I felt<br>[ ] yes, related to time spent seeking healthcare<br>[ ] no, unrelated to my illness<br>[ ] I don't know<br>[ ] my income only changed because I lost/gained a job<br>[ ] N/A	Period 2 is the period between end pf period 1 and start of period 3.
1.7	If there were changes in your personal income level over period 3, **that were not caused by loss/gain of a job**, were they because of your illness - please tick the one that most applies?	[ ] yes, related to how ill/well I felt<br>[ ] yes, related to time spent seeking healthcare<br>[ ] no, unrelated to my illness<br>[ ] I don't know<br>[ ] my income only changed because I lost/gained a job<br>[ ] N/A	
**Personal nonmonetary income**			
2.0	How much have you earned in nonmonetary income (eg. Food) per month at the **start of Period 1** ?	___________ Rand/month OR<br>[ ] nothing OR<br>[ ] don't know	(enter the amount of money that the nonmonetary payments could be sold for at market)
2.1	...and at the **end of period 1?**	___________ Rand/month OR<br>[ ] nothing OR<br>[ ] don't know	
2.2	...and at the start of Period 3?	___________ Rand/month OR<br>[ ] nothing OR<br>[ ] don't know	Skip to 3.0 if no changes in earnings **in any period**
2.3	…and at the end of Period 3?	___________ Rand/month OR<br>[ ] nothing OR<br>[ ] don't know	
2.4	If there were changes in your personal nonmonetary income level over period 1, were they because of your illness - please tick all that apply?	[ ] yes, related to how ill/well I felt<br>[ ] yes, related to time spent seeking healthcare<br>[ ] no, unrelated to my illness<br>[ ] I don't know	

2.5	If there were changes in your personal nonmonetary income level over period 2, were they because of your illness - please tick all that apply?	[ ] yes, related to how ill/well I felt<br>[ ] yes, related to time spent seeking healthcare<br>[ ] no, unrelated to my illness<br>[ ] I don't know	
2.6	If there were changes in your personal nonmonetary income level over period 3, were they because of your illness - please tick all that apply?	[ ] yes, related to how ill/well I felt<br>[ ] yes, related to time spent seeking healthcare<br>[ ] no, unrelated to my illness<br>[ ] I don't know	
**household monetary income, costs of caring**			
3.0	How many members live in your household?	[ ]Adults and [ ]Children	Number of adults (18 years and above) and children (below 18 years)
3.1	Roughly how much monetary income did the people in your household **(excluding you)** gain in total in a month, **excluding remittances or grants**? At the start of Period 1?	__________ Rand/month OR<br>[ ] nothing OR<br>[ ] don't know	remittance= money sent from someone outside the household
3.2	…and the end of period 1?	__________ Rand/month OR<br>[ ] nothing OR<br>[ ] don't know	
3.3	…and at the start of period 3?	__________ Rand/month OR<br>[ ] nothing OR<br>[ ] don't know	
3.4	…and the end of period 3?	__________ Rand/month OR<br>[ ] nothing OR<br>[ ] don't know	
3.5	If there were changes in your household income level (ie. household income **ex**cluding your own) over period 1, were they because of your illness?	[ ] yes<br>[ ] no<br>[ ] don't know<br>[ ] N/A	household income level is personal income + household excluding pt
3.6	In period 1, did an adult in your household spend time looking after you, as a result of your illness?	[ ] yes, ______hours per day, and ______day(s) or month(s) in total<br>[ ] no<br>[ ] don't know	If no skip to 3.7
3.7	In period 1, did an adult in your household have to take over your household tasks, as a result of your illness?	[ ] yes, ______hours per day, and ______day(s) or month(s) in total<br>[ ] no<br>[ ] don't know	If no skip to 3.9
3.8	If there were changes in your household income level over period 2, (ie. household income **ex**cluding your own) were they because of your illness?	[ ] yes<br>[ ] no<br>[ ] don't know<br>[ ] N/A	
3.9	In period 2, did an adult in your household spend time looking after you, as a result of your illness?	[ ] yes, ______hours per day, and ______day(s) or month(s) in total<br>[ ] no<br>[ ] don't know	if no skip to 3.12

3.10	In period 2, did an adult in your household have to take over your household tasks, as a result of your illness?	[ ] yes, ______hours per day, and ______day(s) or month(s) in total<br>[ ] no<br>[ ] don't know	If no skip to 3.14
3.11	If there were changes in your household income level over period 3, (ie. household income **ex**cluding your own) were they because of your illness?	[ ] yes<br>[ ] no<br>[ ] don't know<br>[ ] N/A	
3.12	In period 3, did an adult in your household spend time looking after you, because of your illness?	[ ] yes, ______hours per day, and ______day(s) or month(s) in total<br>[ ] no<br>[ ] don't know	if no skip to 3.17
3.13	In period 3, did an adult in your household have to take over your household tasks, because of your illness?	[ ] yes, ______hours per day, and ______day(s) or month(s) in total<br>[ ] no<br>[ ] don't know	If no skip to 4.0
**Household nonmonetary income**			
4.0	Roughly how much nonmonetary income did your household gain in total in a month (excluding your non-monetary income)? At the start of period 1:	__________ Rand/month OR<br>[ ] nothing OR<br>[ ] don't know	
4.1	…and at the end of period 1?	__________ Rand/month OR<br>[ ] nothing OR<br>[ ] don't know	
4.2	…and at the start of period 3?	__________ Rand/month OR<br>[ ] nothing OR<br>[ ] don't know	
4.3	…and at the end of period 3?	__________ Rand/month OR<br>[ ] nothing OR<br>[ ] don't know	If no changes in any period **skip** to 5.0
4.4	If there were changes in your household nonmonetary income level over period 1, were they because of your illness?	[ ] yes<br>[ ] no<br>[ ] don't know	
4.5	If there were changes in your personal nonmonetary income level over period 2, were they because of your illness?	[ ] yes<br>[ ] no<br>[ ] don't know	
4.6	If there were changes in your personal nonmonetary income level over period 3, were they because of your illness?	[ ] yes<br>[ ] no<br>[ ] don't know	
**Household Remittances**			
5.0	Roughly how much monetary income did the people in your household **(including** you) gain in remittances? At the start of period 1:	__________ Rand/month OR<br>[ ] nothing OR<br>[ ] don't know	ie money sent from someone outside the household
5.1	…at the end of period 1?	__________ Rand/month OR<br>[ ] nothing OR<br>[ ] don't know	
5.2	..at the start of period 3?	__________ Rand/month OR<br>[ ] nothing OR<br>[ ] don't know	

5.3	…at the end of period 3?	__________ Rand/month OR<br>[ ] nothing OR<br>[ ] don't know	
5.4	Roughly how much non-monetary income(eg. food) did the people in your household **(including** you) gain in remittances? At the start of period 1:	__________ Rand/month OR<br>[ ] nothing OR<br>[ ] don't know	ie food sent from someone outside the household
5.5	…at the end of period 1?	__________ Rand/month OR<br>[ ] nothing OR<br>[ ] don't know	
5.6	..at the start of period 3?	__________ Rand/month OR<br>[ ] nothing OR<br>[ ] don't know	
5.7	…at the end of period 3?	__________ Rand/month OR<br>[ ] nothing OR<br>[ ] don't know	
5.8	If there were any changes in remittances over these periods, was this a consequence of your illness?	P1 Y [ ] N [ ] N/A [ ] don't know [ ]<br>P2 Y [ ] N [ ] N/A [ ] don't know [ ]<br>P3 Y [ ] N [ ] N/A [ ] don't know [ ]	Both monetary and non-monetary changes
**Grants**			
6.0	Roughly how much money did your **household** gain in pensions, unemployment pay (including schemes you previously paid into) or government grants, **excluding** those specific for HIV/TB? Start of period 1?	__________ Rand/month OR<br>[ ] nothing OR<br>[ ] don't know	
6.1	...end of period 1?	__________ Rand/month OR<br>[ ] nothing OR<br>[ ] don't know	
6.2	...Start of period 3?	__________ Rand/month OR<br>[ ] nothing OR<br>[ ] don't know	
6.3	...end of period 3?	__________ Rand/month OR<br>[ ] nothing OR<br>[ ] don't know	
6.4	Were any changes in grants a consequence of your illness?	P1 Y [ ] N [ ] N/A [ ] don't know [ ]<br>P2 Y [ ] N [ ] N/A [ ] don't know [ ]<br>P3 Y [ ] N [ ] N/A [ ] don't know [ ]	

7.0	Roughly how much money did you personally gain in illness-specific government grants, eg, those specific for HIV or TB? **Start of period 1?**	__________ Rand/month OR<br>[ ] nothing OR<br>[ ] don't know	
7.1	...end of period 1?	__________ Rand/month OR<br>[ ] nothing OR<br>[ ] don't know	
7.2	...Start of period 3?	__________ Rand/month OR<br>[ ] nothing OR<br>[ ] don't know	
7.3	...end of period 3?	__________ Rand/month OR<br>[ ] nothing OR<br>[ ] don't know	
8.0	Have any charities, religious organisations or NGOs assisted you or your household with goods or money because of your illness, and if so, how much have you received?<br>**Start of period 1?**	__________ Rand/month OR<br>[ ] nothing OR<br>[ ] don't know	
8.1	...end of period 1?	__________ Rand/month OR<br>[ ] nothing OR<br>[ ] don't know	
8.2	...Start of period 3?	__________ Rand/month OR<br>[ ] nothing OR<br>[ ] don't know	
8.3	...end of period 3?	__________ Rand/month OR<br>[ ] nothing OR<br>[ ] don't know	If "nothing" to all questions, skip to 9.0
8.4	Was the charity/NGO assistance **a consequence of your illness?**	start P1 end P1<br>[Y/N/NA] [Y/N/NA]<br>start P3 end P3<br>[Y/N/NA] [Y/N/NA]	delete as appropriate
**Days worked/days off**			
9.0	How many days **did you work** per month at the start of Period 1 (excluding days taken off)?	__________ days OR<br>[ ] no days OR<br>[ ] don't know	NB 5 days/week is 22.5 days per month (multiply days per week by **4.5** to get days per month
9.1	...at the end of Period 1?	__________ days OR<br>[ ] no days OR<br>[ ] don't know	
9.2	...at the start of Period 3?	__________ days OR<br>[ ] no days OR<br>[ ] don't know	
9.3	...at the end of Period 3?	__________ days OR<br>[ ] no days OR<br>[ ] don't know	If not in any paid employment skip to 10.0
9.4	At the start of period 1, how many, if any, days of work per month have you lost on account of **seeking medical care**?	__________ days OR<br>[ ] no days OR<br>[ ] don't know	

9.5	...at the end of Period 1?	__________ days OR<br>[ ] no days OR<br>[ ] don't know	
9.6	...at the start of Period 3?	__________ days OR<br>[ ] no days OR<br>[ ] don't know	
9.7	...at the end of Period 3?	__________ days OR<br>[ ] no days OR<br>[ ] don't know	
9.8	At the start of period 1, how many, if any, days of work per month have you lost on account of **being too sick to work, as a consequence of your illness**?	__________ days OR<br>[ ] no days OR<br>[ ] don't know	
9.9	...at the end of Period 1?	__________ days OR<br>[ ] no days OR<br>[ ] don't know	
9.10	...at the start of Period 3?	__________ days OR<br>[ ] no days OR<br>[ ] don't know	
9.11	...at the end of Period 3?	__________ days OR<br>[ ] no days OR<br>[ ] don't know	
**Personal Employment type**			
10.0	How much have you earned, approximately, in "odd jobs" (or "piece jobs")	start P1: ___________ Rand per month<br>end P1: ___________ Rand per month<br>start P3: ___________ Rand per month<br>end P3: ___________ Rand per month	

	Which different sources of income have **you** had over the 3 periods on the timescale? **(insert a number in the box next to all that apply. Use 1 as the employment that the individual spent the most time doing, with higher numbers indicating a job that the person spent less time doing)**				please insert relevant **numbers** into the boxes
		P1	P2	P3	If periods 1, 2 and 3 all the same, or if has never had income eg. Student/Retired (deliberately stopped or decreased earning)/unemplye
10.1	Employed by government;	[__]	[__]	[__]	
10.2	Employed by private for profit sector;	[__]	[__]	[__]	
10.3	Employed by NGO;	[__]	[__]	[__]	
10.4	Self-employed , no employees	[__]	[__]	[__]	
10.5	Self-employed with employees;	[__]	[__]	[__]	
10.6	Self-employed/from home, farmer/ fishing/ agriculture;	[__]	[__]	[__]	
10.7	Unemployed/looking for work;	[__]	[__]	[__]	
10.8	Retired (deliberately stopped or decreased earning)	[__]	[__]	[__]	
10.9	Pupil/student;	[__]	[__]	[__]	
10.10	Disabled/sick;	[__]	[__]	[__]	

10.11	House Maker (ie. not looking for work, but looking after family and house);	[__] [__] [__]	d, skip to 12.0.<br><br>If no changes in income, also skip to 12.0
10.12	Daily labourer	[__] [__] [__]	
10.13	Other, please specify	________________________	
**Consequences within employment - only answer if ever in employment in P1-3**			If not in any paid employment skip this whole question to 12.0
11.0	If you have an employer, have you had any difficulty with obtaining the amount of **sick leave** you needed from your employer during periods 1-3?	[ ] Yes<br>[ ] No<br>[ ] Don't know	
11.1	If you had an employer but then **lost a job** in P1-3, when was this?	Job loss 1:date to nearest half month<br>___/____/201____<br>Job loss 2: date to nearest half month<br>___/____/201_____<br>[ ] N/A	If did not lose a job, skip to 12.1
11.2	If you had an employer and you lost a job in P1-3, did you decide not to work any more or were you made redundant (fired)?	[ ] I decided to leave<br>[ ] I was made redundant<br>[ ] don't know	
11.3	If you had an employer and you lost/left a job in P1-3, **was this a consequence of your illness?**	job loss 1:<br>[ ] Yes [ ] No [ ] Don't know<br>Job loss 2:<br>[ ] Yes [ ] No [ ] Don't know	
11.4	If you lost a job in P1-3, when did you return to work?	Job return 1:date to nearest half month<br>___/____/201____<br>Job return 2: date to nearest half month<br>___/____/201_____<br>[ ] N/A	
**Personal accommodation**			
12.0	Which of these best decribes your accommodation situation, at the start of period 1? _______________	1= I lived as part of a household or on my own in a house of flat<br>2 =I lived in accommodation but was unsure how long I would be able to stay there (eg. from week to week)<br>3= I had no roof over my head at least some nights<br>4= Don't know	Household = a group of people recognising the same head and eating from the same pot
12.1	...and at the end of period 1? _______		
12.2	...and at the start of Period 3? ________		If answered **2 or 3,** proceed to 12.4, otherwise skip to next section
12.3	...and at the end of Period 3? ________		
12.4	If you answered 2 or 3, where are/were you sleeping?	[ ] at friends' places<br>[ ] at a hostel<br>[ ] with relatives	
	Other____		
12.5	If your accommodation became less secure after the start of period 1, **was this a consequence of your illness?**	[ ] yes<br>[ ] no<br>[ ] don't know	
**FOOD EXPENDITURE**			

1.0	We would like to know if there have been any changes to how much food your household has been able to obtain. At the start of **period 1,** what has been your household's expenditure per month on food (**excluding "special foods")?**	__________ Rand/month OR<br>[ ] nothing OR<br>[ ] don't know	(or if home production, how much would the food your household consumed be able to be sold for at market?)
1.1	…and at the end of period 1?	__________ Rand/month OR<br>[ ] nothing OR<br>[ ] don't know	NB "food" total includes energy foods
1.2	…and at the start of period 3?	__________ Rand/month OR<br>[ ] nothing OR<br>[ ] don't know	
1.3	…and at the end of period 3?	__________ Rand/month OR<br>[ ] nothing OR<br>[ ] don't know	
1.4	If there were changes in food expenditure in period 1, were they a consequence of your illness?	[ ] yes [ ] no<br>[ ] NA [ ] don't know	
1.5	If there were changes in food expenditure in period 2, were they a consequence of your illness?	[ ] yes [ ] no<br>[ ] NA [ ] don't know	
1.6	If there were changes in food expenditure in period 3, were they a consequence of your illness?	[ ] yes [ ] no<br>[ ] NA [ ] don't know	
2.0	How much did you spend, per month, on energy foods, special foods (like milk, meat or vegetables) or vitamins and nutritional supplements at the start of period 1**, as a consequence of your illness?**	__________ Rand/month OR<br>[ ] nothing OR<br>[ ] don't know	**NB these are food that the patient would not have bought if (s)he had been well**
2.1	…and at the end of period 1?	__________ Rand/month OR<br>[ ] nothing OR<br>[ ] don't know	
2.2	…and at the start of period 3?	__________ Rand/month OR<br>[ ] nothing OR<br>[ ] don't know	
2.3	…and at the end of period 3?	__________ Rand/month OR<br>[ ] nothing OR<br>[ ] don't know	
**COPING STRATEGIES**			

1.0	Has there been a time when you or any adult in this household had to skip a meal because there was not enough money to buy food at the start of **period 1**?	[ ] Frequently (more than once a week)<br>[ ] Sometimes (between once a month and once a week)<br>[ ] Occasionally (less than once a month)<br>[ ] Never<br>[ ] Don't know	
1.1	…and at the end of period 1?	[ ] Frequently (more than once a week)<br>[ ] Sometimes (between once a month and once a week)<br>[ ] Occasionally (less than once a month)<br>[ ] Never<br>[ ] Don't know	
1.2	…and at the start of period 3?	[ ] Frequently (more than once a week)<br>[ ] Sometimes (between once a month and once a week)<br>[ ] Occasionally (less than once a month)<br>[ ] Never<br>[ ] Don't know	
1.3	…and at the end of period 3?	[ ] Frequently (more than once a week)<br>[ ] Sometimes (between once a month and once a week)<br>[ ] Occasionally (less than once a month)<br>[ ] Never<br>[ ] Don't know	
2.0	Have you **personally** had to borrow from outside of your household to cover financial hardship (eg from a friend, a bank or a relative?)? If yes, how much did you borrow in total in period 1?	[ ] Yes: _____________Rand<br>[ ] No<br>[ ] Don't know	
2.1	…and in period 2?	[ ] Yes: _____________Rand<br>[ ] No<br>[ ] Don't know	
2.2	…and in period 3?	[ ] Yes: _____________Rand<br>[ ] No<br>[ ] Don't know	If no or "don't know", to all questions 2.0-2.2, skip to question 3.0
2.3	If you answered yes to any of 2.1-2.3, who did you borrow money from?		
	[ ] bank<br>[ ] friend<br>[ ] family member<br>[ ] loan company (eg. Payday loans)<br>[ ] individual "loan sharks"<br>[ ] other: ________________________		

2.4	If you answered that you have taken a loan out, how much did you take out and how much did you need to pay back?<br><br>Loan 1: Taken out: _______________ Pay back:___________________<br>Loan 2: Taken out: _______________ Pay back:___________________<br>Loan 3: Taken out: _______________ Pay back:___________________		
2.5	If you answered that you have taken a loan out, **was this a consequence of your illness?**	loan 1: Y [ ] N [ ] NA [ ]<br>loan 2: Y [ ] N [ ] NA [ ]<br>loan 3: Y [ ] N [ ] NA [ ]	
2.6	Have you been keeping up with the agreed payment schedule?	[ ] Yes:<br>[ ] No<br>[ ] Don't know	
3.0	Have you had to sell anything in order to cover financial hardship? If yes, how much money did you obtain from selling things in total in **period 1**?	___________ Rand OR<br>[ ] didn't sell anything OR<br>[ ] don't know	
3.1	…and in period 2?	___________ Rand OR<br>[ ] didn't sell anything OR<br>[ ] don't know	
3.2	…and in period 3?	___________ Rand OR<br>[ ] didn't sell anything OR<br>[ ] don't know	
4.0	Please tick the boxes for all the time periods where one of these events occurred in your household **as a consequence of your illness:**	P1 P2 P3 No N/A	
	Household member engaging in employment when previously not employed	[__] [__] [__] [__] [__]	If no household members tick NA
4.1	Household member taking on additional work as well as old employment	[__] [__] [__] [__] [__]	
4.2	Household member having to change to a different sort of paid employment?	[__] [__] [__] [__] [__]	
4.3	Child leaving school to care for you	[__] [__] [__] [__] [__]	
4.4	Child leaving school to work	[__] [__] [__] [__] [__]	
4.5	Household member contributing more of their earnings to the household (ie keeping less money for themself) than previously	[__] [__] [__] [__] [__]	
4.6	Relationship changes – if married – has a spousal relationship broken up **as a result of your illnes**s, or has your **spouse decreased or stopped sharing money with you**?	P1 P2 P3 No<br>[__] [__] [__] [__]<br>OR<br>I was not married in this time [__]	**traditional marriages accepted** in the definition

	MANY THANKS FOR COMPLETING THIS QUESTIONNAIRE, WE REALLY APPRECIATE YOU TAKING THE TIME FOR US		
