# Supplementary material for: Measuring income for catastrophic cost estimates: Limitations and policy implications of current approaches
Source: Soc Sci Med. 2018 Oct;215:7–15. doi: 10.1016/j.socscimed.2018.08.041 (PMC6171470; doi:10.1016/j.socscimed.2018.08.041)
Supplement: SUPPLEMENTARY FILE 3_Detailed costs for included and excluded participants [file mmc3.docx]

Supplementary Table 1: Total costs for patients included and excluded from analysis

|  | **Participants included in analysis (n = 66)** | | | **Participants excluded due to missing income data (n = 33)** | | |
| --- | --- | --- | --- | --- | --- | --- |
|  | **Average Number Visits** | **Average Direct Medical Cost** | **Average Direct Non-Medical Cost** | **Average Number Visits** | **Average Direct Medical Cost** | **Average Direct Non-Medical Cost** |
| **Study clinic** | $12.98 | $0.00 | $27.32 | $14.15 | $0.00 | $35.64 |
| **Other clinic** | $0.12 | $0.00 | $0.31 | $0.24 | $0.00 | $0.97 |
| **Pharmacy** | $1.44 | $4.60 | $0.86 | $0.55 | $1.49 | $0.92 |
| **General practitioner** | $0.35 | $7.56 | $0.86 | $0.18 | $2.51 | $0.92 |
| **Hospital-inpatient** | $0.12 | $0.80 | $4.49 | $0.18 | $5.94 | $2.56 |
| **Traditional healer** | $0.21 | $8.95 | $0.69 | $0.33 | $35.54 | $0.78 |
| **Specialist** | $0.57 | $0.57 | $1.19 | $0.25 | $0.25 | $0.39 |
| **Radiologist** | $0.00 | $0.00 | $0.88 | $0.00 | $0.00 | $1.16 |
| **DOTS** | $0.00 | $0.00 | $0.00 | $0.00 | $0.00 | $0.00 |
| **Total** | **$15.80** | **$22.48** | **$36.60** | **$15.89** | **$45.73** | **$43.33** |
