# Supplementary material for: Measuring income for catastrophic cost estimates: Limitations and policy implications of current approaches
Source: Soc Sci Med. 2018 Oct;215:7–15. doi: 10.1016/j.socscimed.2018.08.041 (PMC6171470; doi:10.1016/j.socscimed.2018.08.041)
Supplement: SUPPLEMENTARY FILE 4_MCA results [file mmc4.docx]

# Supplementary file: MCA Results

Table 6 MCA Results

|  | **TBFT Dataset Frequency** | **NIDS Dataset Frequency** | **Dimension 1 Coordinates** | **Contribution** |
| --- | --- | --- | --- | --- |
| **Assets** |  |  |  |  |
| Stove |  |  |  |  |
| Owns a stove | 86% | 16% | 1.05 | 1.1% |
| Does not own a stove | 14% | 84% | -0.20 | 0.2% |
| Satellite television |  |  |  |  |
| Owns a satellite television | 38% | 30% | 1.75 | 5.9% |
| Does not own a satellite television | 62% | 70% | -0.77 | 2.6% |
| DVD player |  |  |  |  |
| Owns a DVD player | 71% | 36% | 1.19 | 3.2% |
| Does not own a DVD player | 29% | 64% | -0.66 | 1.7% |
| Motor car |  |  |  |  |
| Owns a motor car | 18% | 18% | 2.11 | 5.0% |
| Does not own a motor car | 82% | 82% | -0.46 | 1.1% |
| Radio |  |  |  |  |
| Owns a radio | 77% | 63% | 0.44 | 0.7% |
| Does not own a radio | 23% | 37% | -0.74 | 1.3% |
| Television |  |  |  |  |
| Owns a television | 87% | 80% | 0.54 | 1.5% |
| Does not own a television | 13% | 20% | -2.24 | 6.1% |
| Computer |  |  |  |  |
| Owns a computer | 17% | 16% | 2.20 | 4.9% |
| Does not own a computer | 83% | 84% | -0.43 | 1.0% |
| Refrigerator |  |  |  |  |
| Owns a refrigerator | 82% | 76% | 0.64 | 2.0% |
| Does not own a refrigerator | 18% | 24% | -2.08 | 6.3% |
| Cellphone |  |  |  |  |
| Owns a cellphone | 97% | 89% | 0.20 | 0.2% |
| Does not own a cellphone | 3% | 11% | -1.63 | 1.8% |
| Bicycle |  |  |  |  |
| Owns a bicycle | 11% | 8% | 1.76 | 1.5% |
| Does not own a bicycle | 89% | 92% | -0.15 | 0.1% |
| Washing machine |  |  |  |  |
| Owns a washing machine | 36% | 33% | 1.83 | 6.9% |
| Does not own a washing machine | 64% | 67% | -0.91 | 3.5% |
| **Toilet type** |  |  |  |  |
| Flush toilet with onsite disposal (septic tank / soak-away) | 54% | 28% | 1.31 | 2.9% |
| Flush toilet with offsite disposal | 3% | 23% | 1.03 | 1.5% |
| Chemical toilet | 0% | 2% | -1.38 | 0.3% |
| Pit latrine with ventilation pipe (VIP) | 16% | 16% | -1.26 | 1.6% |
| Pit latrine without ventilation pipe | 25% | 25% | -0.93 | 1.3% |
| Bucket toilet | 0% | 3% | -1.54 | 0.5% |
| None | 1% | 3% | -2.67 | 1.4% |
| Other | 0% | 0% | -1.53 | 0.0% |
| **Main Walls Material** |  |  |  |  |
| Mud | 0% | 4% | -2.89 | 1.9% |
| Mud / cement | 6% | 7% | -2.77 | 3.2% |
| Corrugated iron / zinc | 17% | 9% | -1.22 | 0.8% |
| Prefab / wood | 0% | 1% | -0.50 | 0.0% |
| Bare | 14% | 77% | 0.54 | 1.4% |
| Plaster / finished | 60% | 1% | 0.19 | 0.0% |
| Other | 2% | 1% | -1.10 | 0.1% |
| **Main Floors Material** |  |  |  |  |
| Natural floor (earth / sand / dung) | 8% | 9% | -2.35 | 3.2% |
| Rudimentary floor (bare wood planks) | 0% | 39% | -0.68 | 1.1% |
| Finished floor (parquet / polished / tiles / cement / carpet) | 92% | 52% | 0.91 | 2.7% |
| **Dwelling Type** |  |  |  |  |
| Dwelling/house on a separate stand or yard or on farm | 73% | 72% | 0.53 | 1.3% |
| Traditional dwelling / hut made of traditional materials | 1% | 12% | -2.44 | 4.3% |
| Flat or apartment in a block of flats | 1% | 2% | 0.67 | 0.1% |
| Town / cluster / semi-detached house (simplex, duplex) | 0% | 1% | 1.20 | 0.1% |
| Dwelling / house / flat / room in backyard | 6% | 3% | 0.33 | 0.0% |
| Informal dwelling / shack in backyard | 14% | 4% | -1.14 | 0.3% |
| Informal dwelling / shack in informal/ squatter settlement | 4% | 5% | -1.62 | 0.8% |
| Room/flatlet | 2% | 1% | -0.55 | 0.0% |
| Caravan/tent | 0% | 0% | 0.03 | 0.0% |
| Other (specify) | 0% | 0% | -0.65 | 0.0% |
| **Source of water** |  |  |  |  |
| Piped inside dwelling | 37% | 39% | 1.40 | 4.8% |
| Piped inside yard | 44% | 30% | -0.15 | 0.0% |
| Piped inside community stand | 14% | 17% | -1.53 | 2.5% |
| No access to piped water | 1% | 3% | -1.74 | 0.5% |
| Borehole | 2% | 2% | -0.69 | 0.1% |
| Open source | 2% | 7% | -2.32 | 2.3% |
| Other | 0% | 1% | -1.55 | 0.2% |

Table 8 Quintile results from MCA

| **NIDS quintile** | **Number Households**  **(TBFT Dataset)** | **Number Households**  **(NIDS Dataset)** | **Mean annual permanent income per household** | **Standard Error** | **95% Confidence Interval** |
| --- | --- | --- | --- | --- | --- |
| 1 | 9 | 5,007 | $193.30 | 3.89 | $185.68 - $200.93 |
| 2 | 9 | 4,869 | $263.30 | 4.42 | $254.63 - $271.96 |
| 3 | 35 | 4,674 | $339.23 | 4.84 | $329.75 - $348.72 |
| 4 | 24 | 4,272 | $501.82 | 14.58 | $473.25 - $530.4 |
| 5 | 22 | 4,418 | $1,109.88 | 17.46 | $1,075.66 - $1,144.11 |
